# Qualitative study of patients’ and clinicians’ experiences of an educational intervention for warfarin therapy control in atrial fibrillation in Thailand

**DOI:** 10.1136/bmjopen-2024-096490

**Published:** 2025-03-13

**Authors:** Wichuda Jiraporncharoen, Nida Buawangpong, Chaisiri Angkurawaranon, Kate Jolly, G Neil Thomas, Arintaya Phrommintikul, Rungroj Krittayaphong, Surakit Nathishuwan, Gregory YH Lip, Deirdre Lane, Jonathan Mathers, the TREATS-AF consortium

**Affiliations:** 1 Faculty of Medicine, Chiang Mai University, Chiang Mai, Thailand; 2 Chiang Mai University Faculty of Medicine, Chiang Mai, Thailand; 3 Applied Health Sciences, University of Birmingham, Birmingham, UK; 4 Mahidol University Faculty of Medicine Siriraj Hospital, Bangkok, Thailand; 5 Department of Pharmacy, Mahidol University, Salaya, Thailand; 6 Liverpool Centre for Cardiovascular Science, University of Liverpool, Liverpool, Merseyside, UK; 7 University of Liverpool, Liverpool, UK

**Keywords:** QUALITATIVE RESEARCH, Anticoagulation, Health Education

## Abstract

**Abstract:**

**Objectives:**

We aimed to understand the (1) perspectives of patients with atrial fibrilation (AF) regarding their experience and implementation of The SAMe-TT_2_R_2_ score-guided approach in anticoagulant-nave Thai patients with atrial fibrillation (TREATS-AF) educational intervention for warfarin therapy control, including views on cultural transferability to the Thai context, and (2) healthcare professionals’ (HCPs) experience of implementing the intervention.

**Design:**

Qualitative research study.

**Setting:**

Three university hospitals and four tertiary care hospitals in Thailand.

**Participants:**

13 newly diagnosed patients with AF and 13 HCPs delivering the TREATS-AF intervention, an intensive structured educational programme.

**Methods:**

Semistructured interviews. Patient participants were interviewed at two time points: 4 weeks and 6 months after intervention delivery. HCPs were interviewed when they had at least 6 months experience of intervention delivery. A thematic analysis of content was informed by the framework analytical approach.

**Results:**

13 patients and 13 HCPs were interviewed; most were female (73.3% of patients and all HCPs). Mean age was 70 (68–76) and 40 (38–42.5) years for patients and HCPs, respectively. There were four categories related to the experience of the TREATS-AF intervention: (1) key experiences of the educational sessions, (2) core perceptions of the educational materials provided, (3) suggestions for improving the educational materials and session, and (4) behavioural change and self-management influenced by the TREATS-AF intervention.

**Conclusions:**

The TREATS-AF intervention assisted interviewees who were newly diagnosed with AF in preparing themselves with the necessary knowledge and skills to manage their condition. They stated that it increased their confidence in self-management.

For implementation, regionalised Thai-related food and beverages, patients' literacy and family support should be considered, and infrastructure support for widespread use in healthcare settings would be required.

**Trial registeration number:**

TCTR20180711003.

**STRENGTHS AND LIMITATIONS OF THIS STUDY**

- Diverse sample of patients and healthcare professional participants with direct experience of the The SAMe-TT_2_R_2_ score-guided approach in anticoagulant-nave Thai patients with atrial fibrillation intervention in a Thai context.
- Interviews with patient participants at two time points.
- The study did not gather perceptions of the broader multidisciplinary healthcare team.
- The study did not have direct observation of reported behaviour change consequent to the intervention.

## Introduction

 Atrial fibrilation (AF) affects 2–4% of the global adult population, with prevalence increasing.^1 2^ In Thailand, the prevalence of AF is approximately 2.8%^3^ and the rate of AF hospitalisation is high^4^; consequently, the growing burden of AF is also accompanied by increasing healthcare costs.^5^

The main goals of AF treatment are to minimise stroke risk, alleviate symptoms and optimally manage comorbidities. Current clinical guidelines^6 7^ advocate a holistic or integrated care approach to AF management incorporating the ABC pathway,^8^ and adherence with such an approach has been associated with improved clinical outcomes.^9^ Avoidance of stroke with oral anticoagulation (OAC), such as a vitamin K antagonist (VKA), to keep the international normalised ratio (INR) within the therapeutic range of ≥65–70%^10 11^ or use of a non-VKA OAC is an essential component.

Patients’ self-management is crucial to optimise treatment goals, and patients' health literacy is associated with better self-care behaviours and disease outcomes.^12 13^ Additionally, there is growing evidence that implementing health education and enhanced self-monitoring improves disease control greater than usual care alone.^14^ Self-care behaviours such as good medication adherence and lifestyle modification to reduce risk are crucial components of integrated AF management.^7^ In addition, caregivers or family members are a crucial factor in enhancing medical adherence and supporting long-term AF management.^15 16^ A Cochrane review demonstrates that insufficient information exists to draw conclusions regarding the effect of educational or behavioural treatments on the time in therapeutic range (TTR) in patients with AF receiving VKA therapy. A recent systematic review highlighted the evidence to support the cost-effectiveness of anticoagulation service interventions for patients with AF.^17^

Consequently, additional studies are required to evaluate the effect of therapies on anticoagulation management in patients with AF and the mechanisms underlying their efficacy.^14^ The original TREAT (TRial of an Educational intervention on patients’ knowledge of Atrial fibrillation and anticoagulant therapy, INR control, and outcome of Treatment with warfarin) intervention study showed that education helped improve the TTR for patients with AF taking warfarin during the first six months.^18^ Potentially, adverse clinical outcomes could be reduced by enhancing patients' understanding of warfarin’s necessity and treatment risk. Improving the educational opportunities and self-efficacy of patients with AF is essential for ensuring treatment effectiveness.^18^

The SAMe-TT_2_R_2_ score-guided approach in anticoagulant-naive Thai patients with atrial fibrillation (TREATS-AF) programme^19 20^ aimed to improve INR management in anticoagulant-naive patients by providing AF and anticoagulation knowledge and understanding,^18 21^ via a patient-centred intervention addressing AF and its management combining theoretical and clinical frameworks with patient feedback for patients with AF, and self-care advice for patients and caregivers. The SAMe-TT_2_R_2_ score combines demographic and clinical variables to identify the likelihood of achieving optimal TTR.

TREATS-AF is the first health education programme for patients with AF that has been introduced in Thailand. Using qualitative research techniques, we aimed to understand (1) patients’ perspectives regarding their experience and implementation of the TREATS-AF intervention, including views on the cultural transferability of the TREAT intervention to the Thai context, and (2) healthcare professionals’ (HCPs) experience of implementing the TREATS-AF intervention.

## Methods

### TREATS-AF project

The protocol for TREATS-AF, an educational-behavioural change intervention for patients with AF, adapted from the TREAT study, has been published.^19^ In summary, TREATS-AF is a multicomponent intervention including an educational booklet, patient food and lifestyle diary, a worksheet and video to provide and reinforce relevant health information. The educational booklet (online supplemental file 1) covers AF causes and complications, warfarin treatment, risk of stroke or bleeding on treatment and lifestyle advice. The 23-min-long video contains HCPs’ and ‘expert patient’ narratives that cover AF causes, complications, treatment options, warfarin, INR monitoring and lifestyle advice, along with barriers to self-care. The patient worksheets include a simple self-calculation of stroke risk (CHA_2_DS_2_-VASc score), individual barriers to uptake of warfarin and personal goals for lifestyle changes. In addition, a self-monitoring diary, including diet, alcohol intake (in units), warfarin regimen and INR results, is also provided. Patients and caregivers attend a hospital-based training session delivered by a pharmacist or nurse within 4 weeks of initiating warfarin, lasting 60–90 min, at which the different components of TREATS-AF are discussed. The patients receive a take-home educational booklet, diary and a USB with video content. The TREAT intervention^18 21^ was adapted for use in the Thai context, for example, food, vegetables, herbs and alcohol beverages at the start of this project to form the TREATS-AF intervention.

The TREATS-AF trial is a prospective randomised controlled trial examining the impact of the TREATS-AF educational intervention compared with usual care based on a SAMe-TT_2_R_2_ score-guided strategy in anticoagulant-naïve Thai patients with AF.^19^

### Study design

A qualitative research substudy of the TREATS-AF trial (Thai Clinical Trials Registry identification number TCTR20180711003) included patients with AF and staff providing the TREATS-AF intervention. This study is reported according to the Consolidated Criteria for Reporting Qualitative Research guidelines.^22^ Patients were involved in the design stage of the trial, for example, during the intervention adaptation to the Thai context.

### Setting

The qualitative research was undertaken at three university hospitals (Maharaj Nakorn Chiang Mai Hospital, Siriraj Hospital and Queen Sirikit Heart Center of the Northeast) and four additional tertiary care hospitals (Maharat Nakhon Ratchasima Hospital, Chiang Rai Prachanukroh Hospital, Nakornping Hospital and Lampang Hospital) in Thailand. These geographically dispersed hospital settings were selected to provide diversity in participant characteristics (eg, educational attainment and sociodemographics).

### Sampling and recruitment

The TREATS-AF trial includes newly diagnosed patients with AF referred for warfarin therapy. Full inclusion and exclusion criteria are available in the study protocol.^19^

Participants in the qualitative research included patients and HCPs participating in the TREATS-AF trial. Purposive sampling of patient participants aimed to achieve some diversity according to age and educational attainment as these factors may influence engagement with, and experience of, the TREATS-AF educational-behavioural change intervention. Initial sample size targets (n≈15 participants) interviewed at two time points were estimated from previous research experience. Emerging data and analytic themes were judged iteratively alongside data collection at the initial interview, with no new substantive analytic insights emerging from later interviews. Potential participants were provided with written information about the qualitative substudy and invited to participate by local clinical staff. Informed consent was taken prior to the commencement of the initial interview by the project qualitative researcher. Where patient participants requested that a caregiver also attend the interview, this was permitted. In addition, all HCPs from all centres delivering the TREATS-AF intervention (predominantly pharmacists) were invited to participate.

### Data collection

Patient participants were interviewed at two time points: first, within 4 weeks of randomisation to TREATS-AF following the in-person educational session, and second, 6 months after intervention delivery. Each interview, guided by an iteratively developed topic guide (online supplemental files 2-4), lasted approximately 30–40 min and was conducted by one experienced female clinical qualitative researcher from Chiang Mai University (WJ) by telephone. The initial interview focused on patients’ experience of the TREATS-AF intervention, including the in-person session and adjunct resources, and explored the acceptability of the intervention. The second interview explored the integration of the educational-behavioural change intervention and information and subsequent lifestyle changes advised by the TREATs-AF intervention and further examined participants’ perspectives on the utility of different intervention components. Interviews with HCPs were undertaken at one time point only, 6 months after the end of their intervention delivery, and explored their experience with, and implementation of, the TREAT intervention in a Thai context. Interviews were conducted with reference to a semistructured guide that was iteratively refined.

### Data analysis

Data collection and analysis were carried out iteratively. Interviews were audio-recorded and transcribed verbatim for analysis. Interviews were conducted in Thai and then transcribed directly to English for team analysis. Translations were checked for accuracy by Thai members of the research team. A thematic analysis of the interview content was informed by the framework analytical approach.^23^

Data were uploaded into NVivo (V.12) for analysis. Each transcript was read multiple times to aid familiarisation and check the accuracy of each transcript. Initial coding was undertaken by two researchers (WJ and NB) to start the development of the coding framework. Preliminary coding was then discussed among the wider research team (WJ, CA, JM, DL and NB), with subsequent refinement of the framework. Agreed codes were then applied to the entire data set (indexing) prior to further team discussion relating to the core thematic findings. Comparative cross-case analysis aided further analytical insights. The coding framework developed from the patient interviews was also used to start coding the HCPs’ interviews and augmented with new codes as necessary. Core analytical insights were organised according to initial research questions focused on the TREATS-AF intervention.

## Results

### Demographic profile

**Figure 1:**
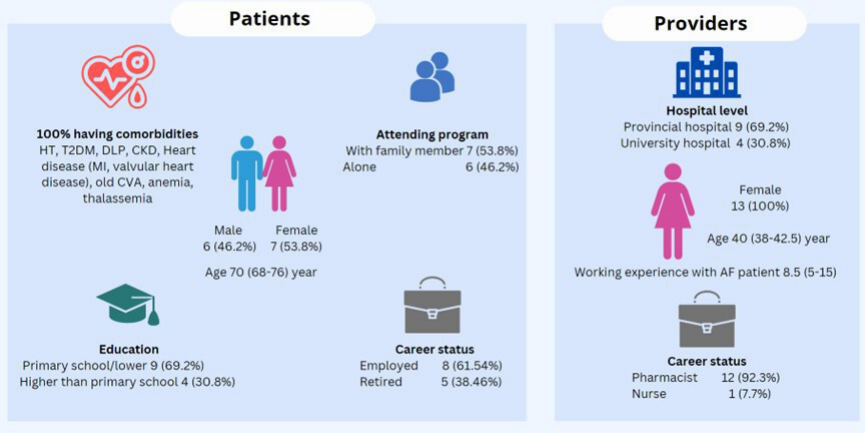
Participant characteristics of patients who attended The SAMe-TT_2_R_2_ score-guided approach in anticoagulant-nave Thai patients with atrial fibrillation (TREATS-AF) intervention and healthcare professionals providing the intervention. CKD, chronic kidney disease; CVA, cerebrovascular accident; DLP, dyslipidaemia; HT, hypertension; T2DM, type 2 diabetes mellitus.

26 participants were interviewed, 13 patients and 13 HCPs, most were female (73.3% of patients and 100% of HCPs) (figure 1). The median (IQR age was 70 (68-76) and 40 (38–42.5) years old for patients and HCPs), respectively. All patients had one or more comorbidities, and most had an educational attainment level of primary school or lower (n=9, 69.2%) and were employed (n=8, 61.5%). Seven patients (53.9%) attended the one-off educational session with a family member. Most of the HCPs were pharmacists (n=12, 92.3%) and worked in provincial hospitals (69.2%), and the median (IQR) time working with patients with AF was 8.5 (5-15) years.

### Themes and subthemes

**Figure 2:**
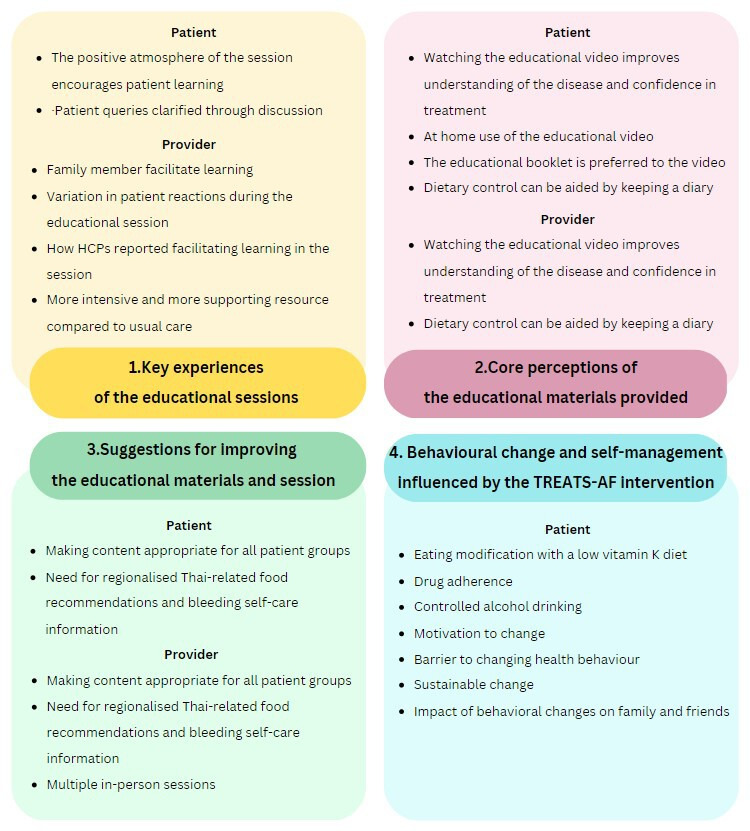
Summary of the themes and subthemes from patients and healthcare professionals (HCPs). TREATS-AF, The SAMe-TT_2_R_2_ score-guided approach in anticoagulant-nave Thai patients with atrial fibrillation.

From our analysis, we present four categories of findings related to the experience of the TREATS-AF educational intervention and its perceived influence on health-related behaviours: (1) key experiences of the educational sessions, (2) core perceptions of the educational materials provided, (3) suggestions for improving the educational materials and session and (4) behavioural change and self-management influenced by the TREATS-AF intervention. Data relating to the first three categories were derived from both the patient and HCP interviews. Themes relevant to the fourth category were derived from patient interviews only. The details of the subthemes are presented in figure 2.

#### Key experiences of the educational sessions

Patients stated that they responded positively to the learning programme because of the supportive and informative learning atmosphere of the session. They highlighted the opportunity to clarify any uncertainties through discussion with the healthcare team and the chance to ask questions. HCPs compared the programme’s perceived strength to usual care, such as opportunities for more intensive interaction with patients, and more supportive resources for these interactions. Additionally, HCP interviewees also talked about responding to both verbal and non-verbal patient reactions in sessions in order to stimulate learning when patients and families were struggling to understand. There was consensus from the interviews that the sessions tended to facilitate high-quality interactions between patients, carers and HCPs compared with usual care.

##### Positive atmosphere of the session encourages patient learning

Most patients found that the atmosphere of the session encouraged their learning. They felt comfortable with the friendly approach from the healthcare team.

*I think it was so good and comfortable. The doctor’s team didn’t put any pressure on the class, and they explained everything well and easy to understand.* (Patient 9)

##### Patient queries clarified through discussion

Most of the patients stated that they enjoyed talking with, and questioning, the HCP about topics such as medication, diet and alcohol consumption. They also liked the opportunity for clarification from the HCP on queries arising from the other intervention materials, such as the video and booklet.

*“It’s what I liked when the pharmacist came because the videos could not complete my understanding. But when there was someone like the pharmacist, it was two-way communication. We got questions and answers.”* (Patient 3)

##### Family members facilitate learning

Almost all of the HCPs commented that the inclusion of family members in the training session was advantageous to the patients’ learning and care. Family members aided patients who struggled with reading and writing, such as older patients or those with low literacy levels. This helped them receive and understand information on the self-care required for optimal AF management.

*Most of them come with family because they are elderly people. It seems that their families make it easier to advise elderly patients as they help to remember the information. Also, their families see the importance and help take better care of the patients. That’s my opinion because if their families are educated, they will know how important the advice is to the patients.”* (HCP 401)

##### Variation in patient reactions during the educational session

Most patients stated that they and their relatives responded positively and were able to comprehend the range of educational materials. HCPs remarked how they nodded, asked further questions or noted the key aspects of the session.

*They paid attention and took notes. For example, if there were questions, they asked. And which foods will cause problems and they wrote down in the handbook. They seem to have responded well.* (HCP 601)

However, there was some variation in reactions to each activity, with less positive feedback from some patient participants concerning boredom from lengthy learning, worries induced by the high score of the stroke risk assessment or their inability to make appropriate meal choices such as vitamin K level in each meal. It was clear from patient and HCP interviews that for some the content of the session was considered too intellectual with lots of medical terminology, which some patients did not understand, and some seemed confused by.

*I do not understand some words in handbook. There are medical terms. If you use a medical term, you should have its meaning.* (Patient 7)

*Some get confused, and I need to explain more. Some local people do not understand big, formal words.* (HCP 502)

##### How HCPs reported facilitating learning in the session

HCPs discussed how they would help patients and family members if they appeared to be having trouble understanding content, for example, by offering pauses in the video with further explanation and pointing out connections between the problem and the patient’s symptoms or behaviour. While the amount of time spent helping was reported to vary, it was noted that the session could be challenging for older people. Additionally, HCP interviewees talked about employing simple language for explanation.

*They will express it through their gestures or words. So, until the content we teach is covered, we try to keep engaging our patients in the conversation. We try to relate it to them to attract their attention, like talking about symptoms. If the patients have those symptoms, they will respond to us. We try to create interaction as much as possible, and there are some questions about risks.* (HCP 102)

##### More intensive and supportive resources compared with usual care

HCP interviewees identified two main ways in which the educational session differed from the interactions with patients in usual care: (1) the intervention training included more self-care materials (video, booklet and diary) and more detailed information such as descriptions of the disease, medication (specifically warfarin) and interactions of, and understanding of, the impact of eating and drinking habits on INR control; (2) the educational session increased patient and family participation in self-care training activities such as self-risk assessment, understanding their concerns and knowledge enquiry. HCP participants stated that the TREATS-AF intervention offers more education than usual, more in-depth information and a greater variety of teaching activities. While this was generally talked about in a positive manner, it did require training, longer interactions with patients and additional spaces (training room). It was also added to staff duties. It was felt that it could affect service capacity, if not resourced and the system is not prepared, and that it would require more staff for routine implementation. Interviewees also stated that there is no established protocol for patients to be followed up in usual care, and the educator may be a general pharmacist rather than a specialist in AF medication.

*I think the project gives a lot of details in deeper information and it makes patients come to the point where they understand and realize the importance of their behavior and taking medicine. Also, practical activities, this is like motivating them to monitor themselves. I think this tends to be better. Only it will affect the service processing because this takes a longer time. If we apply this, we will need more staff in charge and a separate section.* (HCP 601)

### Core perceptions of the educational materials provided

TREATS-AF used some educational materials in the session and to take home. A video was played during the session to share patient experiences with AF and educate about the disease and treatment options. Both patients and HCPs found that the video assisted in understanding the disease and confidence in treatment during the sessions. For the booklet, HCPs used it to communicate with the patients about disease, treatment, self-care behaviours and recording diaries, and patients stated that the booklet was useful for their self-care by providing the opportunity to review information and record eating behaviours. However, some HCPs found that some of the content, such as the type of disease in the booklet, was difficult for people to understand, and its design or presentation was not friendly to older patients such as small text size, a lot of text and few pictures.

*Most importantly they seem to put attention to the part where other patients shared their experiences. Within the family during watching, they talk and share what can be eaten and what kind of activities they can do. This helps release their stress and is willing to take the medicine*. (HCP501)

*What benefits patients is probably the knowledge in the video. It should help them understand the disease better. They know more, and some of them ask more detailed questions.* (HCP301)

*Like types of Atrial Fibrillation, which, in fact, are Persistent AF, Paroxysmal AF, Permanent AF. The book explains the definitions, but the patients might not need to categorize themselves. It should give broad information instead. It’s not a problem for them to take medicine even if they don’t know this. And the other parts that explain the mechanism of action are too deep*. (HCP101)

*And the risks of blood clots in the brain are presented in a pie chart, showing the percentage. This can be understood by only some patients. Those who have never learned about graphs find it hard to understand*. (HCP101)

#### Watching the educational video improves understanding of the disease and confidence in treatment

Most patients discussed liking the inclusion of patient experiences in the video. This section helped them understand self-care, the disease and disease consequences.

*It told how to behave. There were 2 patients at different ages: A male teacher and a lady. They described what they did so that I could follow. I felt that they gave me insight as to how I should behave. It’s the first step that made me confident about the treatment.* (Patient 6)

However, a few patient participants could not remember the video content. HCPs thought that the video would lower patient anxiety about the side effects of medication as it also gave better understanding of the disease, treatment and self-care for patients. They stated that the video makes it easier for less-well-educated and older patients to access this information and attain this knowledge. However, the video’s limitations include a long duration, the use of academic vocabulary and medical terms, which might be difficult for some older patients and those with lower educational attainment. Therefore, it required additional explanation from HCPs during the session.

**Some***lose focus in the first 5 minutes, while some others listen to the end. But from my interviews with patients, I feel that they learn well. My understanding is that they feel they have the same symptoms (as the patient in the video). Probably, patients understand and learn well. When I talk to them, I will see if they can answer (questions) or learn anything.* (HCP301)

#### Use of the educational video at home

Before providing a USB (a drive containing the VDO material), some HCPs talked about evaluating the patient’s need and ability to use a video at home, including a suitable device for playback and family members who can help. Some HCPs had modified the video by converting them into QR codes and sharing them on other social media. Some patients did not want to take the video as they stated that they did not have a suitable device to watch it on at home:

*I don’t have a chance to watch it yet because I have to wait for my son to do it. I don’t know how to use the video player, so my son will do it on Saturday or Sunday.* (Patient 2)

*As for the video, I don’t give them the thumb drive, but I ask everyone whether they want it or not. I can give it, but the patients say they understand after watching it one time. They won’t re-watch because they don’t take the video home.* (HCP 201)

#### Educational booklet is preferred to the video for use at home

Unlike the video, most patients read the booklet at home. They felt that books are practical and convenient because they are a simple way to find the answer, available at any time and easy to review. Again a few interviewees had complaints about overly technical terms, which made it difficult to understand. In some cases, there was a suggestion for pictures to assist in understanding as a few interviewees reported that they did not read and needed a family member to read for them.

Some HCPs regarded the booklet as a knowledge resource that patients can access at any place and any time. However, they identified some similar limitations to patient participants, in that the information about the disease was too difficult for their patients. The contents of the book were talked about as difficult for some patients, such as drug tables, graphs, medical terminology and strange words as a result of direct translation from English.

*I have to read it multiple times and summarize in my own words, making it short and easy to put into practice. Doctors’ language is hard to understand. I try to understand it in my own way. What I should do, causes of the disease, food to be avoided, I read all about these in the handbook and jot down*. (Patient 6)

There were also some criticisms of the formatting of the written materials, such as too much text, not enough photos and the font size being too small. Some older patients and those with poor levels of education could not use it due to these obstacles. Some HCPs reported using additional media when explaining the booklet to make it easier to understand, such as the warfarin book (from a pharmaceutical company) and downloadable applications that assess the risk of stroke. They thought that relatives could help patients understand during the educational session by explaining and taking notes instead of the patient, especially for older patients.

#### Dietary control can be aided by keeping a diary

Most patients reported recording their food diaries as the programme suggested. They felt it assisted their self-control and was used for discussion during follow-up with the healthcare team. HCPs perceived that the advantage of the diary was that the patient has seen what he or she ate throughout the recording period, and if there is a problem, it can be reviewed with the doctor. Patients can write down problems or concerns while they are at home, giving the HCP an overview of information when they meet patients.

*With the diary, we can review what we have eaten, and so can the doctors and the pharmacists. That allows us to discuss and learn more. The doctors give advice about diet, behaviors, and diseases. We can ask them”*. (Patient 6)

However, recording was also difficult because the font size and the lines in the diary were relatively small, as was the instructional text, and patients noted that there were no examples of what to write, to follow. Along with the unfamiliarity of keeping a diary, patients must possess reading and writing skills.

Most HCPs felt that patients need support from family because most are older and have some limitations for diary completion. However, at the time of the interview, HCPs had not yet reviewed diaries with patients to confirm this and the amount of reporting.

*If they are elderly people, they will need help from their relatives. Those patients, elderly uncles, and aunts, they cannot read well because the fonts are small. So, relatives need to help and explain each area. Plus, the daily notetaking in the booklet, the blank space is small but elderly wrote big, this space may need to add.* (HCP 302)

### Suggestions for improving the educational materials and session

Around half of the patients stated that all the educational materials were already good enough; however, as already noted, a few pointed out the limitations of their design or presentation for older patients. HCPs gave suggestions for improvements in both content and presentation. Subthemes in this category include making content appropriate for all patient groups, the need for regionalised Thai-related food recommendations and bleeding self-care information and the option for multiple in-person sessions.

#### Making content appropriate for all patient groups

Participants felt that the educational content in the booklet and video could be made easier for all by removing some complex content, adding more pictures and avoiding or explaining medical terms. It was noted by patients and HCPs that the presentation could be better adapted for older patients, such as increasing the size of the font and spaces for writing, providing details on the order of recording, providing examples and making the videos more attractive, by shortening or improving the delivery of the material.

*We should try to shorten the video to attract more attention from the patients. What we have to do is to hire professionals to make it more attractive, exciting, fascinating so that they want to watch it again.* (Patient 1)

*I just want to have bigger tables, bigger texts, because most readers are elderly, and they have eyesight problems and use easy-to-understand language, not complicated vocabulary. (Booklet).* (Patient 5)

#### Need for regionalised Thai-related food recommendations and bleeding self-care information

Participants recommended that the educational booklet should be more contextualised for Thailand, particularly by region, as, for example, there is variation in local food and vegetables. Some people living in northern or northeastern parts are not familiar with the food and vegetables in the central region. Although the content in the booklet was translated in Thai and added common types of food and vegetable, there are various types of local vegetable in different parts of Thailand which are not mentioned in the booklet.

*In part of food, I’m not sure if this topic was translated from a foreign language because we don’t have some vegetables mentioned in the book in our country. And there are not include our local vegetables such as parsley”* (HCP 302)

Additionally, participants thought that the inclusion of basic bleeding self-care information would be useful.

#### Multiple in-person sessions

The general suggestions for implementing in clinical practice include increasing the number of sessions to better understand and educate patients more about the problems they face when they return home. Participants thought each session could then be more concise and tailored to the clinical context within which the training was being delivered. This would ensure that patients are continuously emphasised and followed up.

*If we will apply and use this educational programme in reality. We would meet with the patients continuously and we can keep telling and emphasise the information in each visit better than giving a ton of details the first time.* (HCP 601)

### Behavioural change and self-management influenced by the TREATS-AF intervention

At the follow-up interview, patients reported health behaviour changes and self-management influences resulting from the TREATS-AF intervention, including dietary modifications, for example, adhering to a low vitamin K diet and controlling alcohol consumption, and improved drug adherence. The motivation to change was driven by their family’s support and their own concern about the disease. A few barriers to behavioural change were identified by patients during the interview, such as symptoms after stopping alcohol or not knowing the level of vitamin K in different types of vegetable. There was some impact from behavioural change on family and friends in terms of support and relationships.

#### Dietary modifications with a low vitamin K diet

Almost all patients stated that they had changed their eating behaviours. The majority kept their high vitamin K diet under control by reducing meal portion sizes or frequency of eating. Some reported avoiding eating foods with high vitamin K content by cooking at home and using food recommendations from the TREATS-AF programme. One participant shared that she would check for ‘risky’ foods before eating it. However, there was evidence of a misunderstanding from one participant, who stated that they ate a high vitamin K diet at a different time from taking warfarin in order to prevent food-drug interactions. A few interviewees had family members remind them about dietary modifications, predominantly related to high vitamin K content foods. About half of the participants demonstrated their ability to self-manage by explaining in detail how they dealt with behavioural issues.

*Collard greens, gourd, climbing wattle, and moringa are in the guidebook that I read. I avoid these vegetables. I eat a little once in a while.* (Patient 5)

### Drug adherence

In interviews, only one patient participant talked about the ways to reduce errors when taking warfarin, which is a complex regimen (different doses on different days). She connected this to the educational intervention which helped remind her by making a note and highlighting the drug regimen.

*I must take the medicines as the doctor tells me. For example, there are medicines that the doctor highlights. This one must be taken half a pill per time, that one from Monday to Thursday. I make reminders about them. This one must be taken before meals, I highlight. The doctor tells me to take less of that one, I make another reminder about it. It reduces my errors*.” (Patient 5)

### Controlled alcohol drinking

A small number of patients who reported drinking alcohol tried to change their drinking habits. One stopped drinking, while the others reduced their alcohol consumption in accordance with the advice from TREATS-AF. The strategies to change alcohol consumption include controlling socialising, going to bed early and telling people around them about their health problem and the need to limit their alcohol consumption as a result.

*I changed my activity as not often hang out and reduce the amount of the drink. I go to bed earlier, previously I went to bed around 3-4 a.m.* (Patient 1)

*I totally stop it. My children and husband know this, too. The doctor does not allow this. I still join the party, but don’t drink.* (Patient 6)

### Motivation to change

Most of the participants stated that behaviour change was driven by their own concerns: being afraid of having more severe problems and being burdensome to their family.

*I must take care of myself if I do not want to be sick, because being sick makes others burdensome.* (Patient 7)

Interviewees talked about not wanting to have critical care at a hospital or being a burden to families if the disease got worse. Some talked about the support from family, while family members said that they desired to take care of their parents as well as they could. Some interviewees also mentioned that receiving good advice from their healthcare team influenced their change.

### Barriers to changing health behaviours

There were stated barriers to reducing or stopping alcohol, namely alcohol withdrawal symptoms in those with chronic alcohol drinking and having fewer relationships with friends with whom they socialised with over alcohol.

*I try not to drink alcohol because when I continue having it for many days it makes me feel so tired. But when I don't have it, I cannot sleep. I only sleep for a couple hours, and then awake until morning.* (Patient 1)

Understanding which foods are high in vitamin K, to enable limiting their intake (rather than avoidance), was perceived to be difficult because, as noted already, the dietary information did not list all common Thai vegetables. In addition, one patient felt that some kind of vegetables (eg, garlic) was a basic ingredient of Thai food, so it was often difficult to avoid high vitamin K vegetables. All participants felt that money and the COVID-19 situation were not barriers to changing health behaviours as local vegetables were readily available. Most patient participants were older and had already spent time at home or living in rural areas, so the pandemic had little effect on changing behaviour and daily life, except that some could not attend the hospital for INR checks during this time.

### Sustainable change

After follow-up at 6 months, all participants stated that they had still maintained the required changes to their behaviour to benefit their health, especially dietary modifications. Most participants were able to explain in detail the diets they follow, and to manage their diet by themselves, choose what to eat from the food prepared by others (such as choosing the right food or avoiding prohibited food) or preparing the food themselves. Only one participant who had had a stroke was supported by her family in this regard. All the participants who had been drinking stated that they still continued to reduce their drinking as recommended. A few participants mentioned self-management in terms of preparing medication and continuing to take it on a regular basis.

*Taking warfarin, I avoid broccoli and leafy green vegetables. I rarely have those myself. I do it every day, avoiding certain kinds of vegetables which I am not allowed to eat.”* (Patient 2)

### Impact of behavioural change on family and friends

Most patients thought that there was no effect of their behavioural change (low vitamin K diet control) on their family because they had eaten separately or their families had adjusted by eating the same food with them. However, their family and friends assisted in controlling behavioural change by reminding them to take medication or arranged medication, reminding them to take a low vitamin K diet, recording food diaries and preparing food. When they changed their drinking habits, some participants were concerned that they would have fewer relationships with their friends.

*I got advice and food from my daughter. Moreover, she takes care of my health even if we are not in the same house.* (Patient 10)

*There is some impact because our team of around 7-10 persons work with each other as a family member. After finished work, we would get a meal and maybe drink a little bit. Currently, when I finished the meal, I would come back and did not drink. This is not bad, but my relationship with old friends (i.e., primary friends, high school friends, and the classmate who lived far away) is not good. Previously, we would meet 1-2 times a month.* (Patient 1)

## Discussion

This study has illustrated patients’ and HCPs’ perceptions towards the TREATS-AF intervention and explored patient’s stated behaviour changes and the influence of the intervention on these. Their perspectives were consistent, and they reported that the programme improved AF knowledge, awareness and self-care. In general, the intensive educational programme and the educational materials were perceived positively. Some participants had difficulty in reading or writing due to being illiterate or older and were assisted by HCPs who delivered the content in more simple terms or with further explanation, or alternatively participants were facilitated by family members.

The TREATS-AF intervention is an intensive training programme, and therefore requires additional supportive resources than usual care. Cultural transferability could be enhanced by adding regionalised Thai-related foods, such as vegetables, or alcoholic beverages, and making content appropriate for all patient groups, for example, those from different educational backgrounds. Patients described changing their behaviours in response to the TREATS-AF intervention information, such as dietary modifications with a low vitamin K diet, improved medication adherence and controlling alcohol drinking or abstinence. Family support and patient’s awareness both served as motivators for behaviour modification. However, some barriers to changing health behaviours were reported, including insomnia from alcohol withdrawal, lost friendships due to alcohol cessation and not recognising which elements of the diet should be limited because of high vitamin K content.

The TREATS-AF intervention was acceptable and perceived by both patients and HCPs to improve patient knowledge and assist in self-management for new patients with AF. This structured educational programme provided a comprehensive learning model; basic knowledge of the disease and treatment, sharing experiences of other patients living with AF and discussion of a self-management plan with the healthcare team.^19^ In this sample, the educational materials, written information or video, accompanied by verbal advice, were thought to enhance patients’ health education.^24^ The previous TREAT intervention, conducted in the UK, demonstrated improvements in anticoagulation control in the first six months with an educational-behavioural intervention compared with usual care, and there was an improvement in AF knowledge over time in both groups.^18^

The TREATS-AF intervention provided AF treatment-specific knowledge that improved patients’ understanding and enhanced their awareness of the need for behavioural change which is an important step in self-management.^25^ In a previous qualitative study, patients with symptomatic AF who were interviewed to design an educational programme indicated that they were interested in enhancing their self-efficacy for self-management of symptoms.^26^ When patients understand better their health condition, they are more likely to take an active role in their healthcare, which can lead to improved health outcomes. The key educational messages for self-management of AF should include how prescribed medications, such as warfarin work, how patients should monitor their AF symptoms and be aware of signs/symptoms of bleeding, and how to create an action plan for their care.^12^

In this study, the interviews demonstrate that the TREATS-AF intervention has the potential to impact behaviour by promoting self-efficacy. Patients reported that participation in the intervention helped decrease the stress they felt about having AF and improved their confidence in managing their AF. The sharing of experiences by other patients with AF included in the video helped participants better understand their AF and how to deal with it. Self-efficacy is important in behaviour control and occurs when patients believe that they can overcome the potential barriers to a self-care task.^27 28^

The strength of this qualitative study is that it included both patients and HCP participants with direct experience of the TREATS-AF intervention in a Thai context, thereby providing multiple perspectives relevant to the implementation of this type of educational intervention.^29^ Participants were included from various hospital settings (three university hospitals and four tertiary care hospitals) and included a mixture of patients of different ages and educational levels, and a range of HCPs all with direct experience of delivering the intervention. There are also some limitations. First, the perception of HCPs was limited to the view of pharmacists, and not multidisciplinary teams. However, this is reflective of the HCPs who would normally deliver information about OAC to patients with AF in Thailand. It is possible that other groups of HCPs may hold different views about the utility, content and delivery of the intervention. Most pharmacists in Thailand are female, hence the gender distribution observed in our provider sample. Second, the interview was conducted via Zoom, online video conferencing, as necessitated by the COVID-19 pandemic. This may have been unfamiliar to some older people when communicating and may have limited the interaction and rapport between the patient and interviewer and consequently the information disclosed. However, the people participating were able to engage with the interview as a data collection method that allowed open, in-depth conversation, and critical reflection on the TREATS-AF intervention. We have not observed actual behaviour change in this study and are reliant on self-report during the qualitative research interviews. We have not interviewed participants in the comparator group of the TREATS-AF trial and therefore cannot comment on relative accounts of behaviour change and associated influences for those who did not receive the educational intervention.

For sustainable implementation, various aspects of the TREATS-AF intervention need to be considered. An important behaviour modification required for the management of good INR control is dietary changes, which must consider social influences on eating habits.^30^ Interventions that aim to improve self-care for older people need to consider the possible barriers, including both individual and environmental factors.^31^ Health education for older people needs to be simple, use key messages and limit the use of technical terms.^32^ In this respect, family plays an important role in healthcare, especially for older people. Incorporating family or caregiver assistance is crucial for implementation and maintenance of health behaviours because family members assist patients in changing their lifestyles.^33^ Additionally, they can assist patients with AF in handling difficult situations when they encounter them.^16^ Healthcare delivery involves a complex and dynamic system that requires coordination and collaboration. Effective communication, shared decision-making and mutual respect are essential components of teamwork in healthcare.^34^ Therefore, high-quality healthcare delivery is possible when HCPs, patients and their families work together as a team. The educational intervention should be tailored cognisant of the patient’s and family’s conditions. Another issue that HCPs should be aware of is the patients’ alcohol consumption as some patients may have alcohol dependence or addiction. Providing advice and health education for alcohol cessation may take several steps and continue to be done gradually to prevent alcohol withdrawal symptoms.^35^

Further fine-tuning of the intervention is also required, including more cultural adaptation (eg, inclusion of regional variation in food and alcohol advice), less medical jargon and alternative means of communicating to less literate patient populations and simplified formatting (text, etc) and incorporation of information on the non-vitamin antagonist oral anticoagulants which are becoming more widely available as a treatment option in Thailand.

## Conclusion

The TREATS-AF intervention has the potential to help newly diagnosed patients with AF prepare themselves for living with AF and may help enhance self-efficacy. To improve further implementation, the intervention materials require some revision to include regionalised dietary information, and some tailoring based on patients’ literacy and the availability of family support needs to be considered. The educational intervention needs to be incorporated into existing healthcare services and is therefore reliant on the availability of resources including the infrastructure and trained staff to scale it up.

## supplementary material

10.1136/bmjopen-2024-096490 online supplemental file 1

10.1136/bmjopen-2024-096490 online supplemental file 2

10.1136/bmjopen-2024-096490 online supplemental file 3

10.1136/bmjopen-2024-096490 online supplemental file 4

## Data Availability

Data are available upon reasonable request.
