# Supplementary material for: Qualitative study of patients’ and clinicians’ experiences of an educational intervention for warfarin therapy control in atrial fibrillation in Thailand
Source: BMJ Open. 2025 Mar 13;15(3):e096490. doi: 10.1136/bmjopen-2024-096490 (PMC11907032; doi:10.1136/bmjopen-2024-096490)
Supplement: online supplemental file 1 [file bmjopen-15-3-s001.docx]

Interview Schedule – Timepoint 1 (within 4 weeks of intervention delivery)

*Introduction and opening remarks*

- Researcher introduces self and re-checks practicalities related to the interview (time available, privacy, likelihood of interruptions)
- Achieve informed consent - take participant through information sheet and consent form, ensuring participant is aware of the broad aims of the study, and their rights as research participants. Answer any questions. Ensure consent form is completed and signed by participant and researcher. Fill in consent form on participants’ behalf if undertaken as a telephone interview and verbal consent is being taken. Commence audio-recording once consent is taken.
- Set expectations – (a) Briefly give an overview of the topics that will be discussed during the interview, and (b) explain that we are interested in the participant’s own views on the topics being discussed and that any thoughts and ideas that they have are valued and relevant to the study. There are no right or wrong answers.
- Ensure the participant is comfortable and happy to begin the interview.

**PLEASE NOTE: questions and prompts below are intended to be used flexibly dependent on the interviewee, their responses, and the context of the interview.**

*Background of participant, context, ice breaker*

1. We’re really grateful that you have decided to take part in an interview for this study. What was it that interested you in talking to us about the study as part of a research interview?
2. Could you tell me a little bit about you – whether you have family, where you live etc?

*Background – AF, symptoms, impact, diagnosis, understanding (attempting to provide context relating to the participant’s experience of AF, any AF related symptoms, the impact of these to date (health and broader QoL related issues), how AF was diagnosed, and also their understanding of AF and treatment matters including warfarin therapy).*

1. When did you first start to notice there may be issues with your health related to the AF?
2. Did you have any symptoms from your AF? If yes, what symptoms did you notice? Probe – palpitations, shortness of breath, blackouts/LOC, dizziness etc.
3. How did these symptoms develop? How long ago was that?
4. Did these symptoms impact upon your daily life? If so at what point and how? – Probe around social life, relationships, working life, activities of daily living
5. At what point did you decide to seek medical help?
6. What motivated you to make the decision to seek medical help?
7. When were you diagnosed with AF?
8. What do you understand AF to be? How much do you know about AF? Probe – what is it; what causes it; how does it impact on patients?
9. What do you understand about the treatments available for AF? Probe – what options are available; what are they aiming to achieve; how will they affect your symptoms;
10. Understanding of warfarin therapy – what it consists of; what it aims to achieve; processes and monitoring associated with therapy; factors inc. lifestyle that influence the effectiveness of warfarin therapy; are they aware of what an INR target is and if so what their INR target is; how good is their INR control?

*Recruitment to TREATS-AF and knowledge of the TREATS-AF intervention (section aiming to explore motivations for taking part in TREATS-AF; how they were approached about the study; their understanding of the study; their experience and views regarding the TREAT intervention; their understanding of what they have been asked to do and what if any behaviours they have modified in response to the intervention; how this has impacted on them and those around them; their expectations for the remainder of the study period).*

1. How did you become interested in taking part in the TREATS-AF study? – Probe around how they found out about the study and what motivated them to take part (Family advice, Clinician advice and/or Personal beliefs)
2. Who introduced you to the study? What were you told about the study? Was this information useful?
3. Can you tell me what you think the aims of the study are?
4. Can you tell me what you think the TREATS-AF programme is trying to achieve (if not explored above)?
5. Can you describe what happened on the day of the TREATS-AF session? Probe – what was discussed, who delivered the day, what other people were there, what was it like for you, what did you think about it?
6. What would you say are the good things about it?
7. Would you say there are any bad things about it?
8. What do you think of the resources that you are asked to use at home e.g. DVD?
9. What sort of things are you being asked to do as part of TREATS-AF? Does this mean that you are having to change some things e.g. lifestyle? If so, what are they? How do you feel about that?
10. What have you done differently since the TREATS-AF session? Why? How has that been for you?
11. Has TREATS-AF impacted on anyone else who is close to you? If so how? What implications does it have for them? How do they feel about this?
12. Is there anything that you haven’t done differently since the session that you were advised to consider? If so, why not?
13. What do you intend to do over the next few months after attending the TREATS-AF session?
14. What do you expect will happen as part of the TREATS-AF study?
15. How would you know that TREATS-AF is working for you?

*Closing*

1. That’s all the questions I had for you. Before I turn the recorder off, is there anything I haven’t covered that you would like to discuss?

*Closing comments*

- Switch off recorder
- Thank participant for time and ideas
- Re-iterate confidentiality
- Remind them of the follow up interview and check best method to contact for arrangements for this. Indicate when and how you will be in touch to arrange the follow up interview.
