# Supplementary material for: Qualitative study of patients’ and clinicians’ experiences of an educational intervention for warfarin therapy control in atrial fibrillation in Thailand
Source: BMJ Open. 2025 Mar 13;15(3):e096490. doi: 10.1136/bmjopen-2024-096490 (PMC11907032; doi:10.1136/bmjopen-2024-096490)
Supplement: online supplemental file 2 [file bmjopen-15-3-s002.docx]

Interview Schedule – Timepoint 2 (6 months follow up)

*Introduction and opening remarks*

- Researcher re-checks practicalities related to the interview (time available, privacy, likelihood of interruptions)
- Researcher reminds participant of project and basis of informed consent – free to withdraw at any point etc.
- Set expectations – (a) Briefly give an overview of the topics that will be discussed during the interview, and (b) explain that we are interested in the participant’s own views on the topics being discussed and that any thoughts and ideas that they have are valued and relevant to the study. There are no right or wrong answers.
- Ensure the participant is comfortable and happy to begin the interview.

**PLEASE NOTE: questions and prompts below are intended to be used flexibly dependent on the interviewee, their responses, and the context of the interview. They may change or be added to based on the interviewee’s responses at the T1 interview – specific prompts based on these may be developed for individual interviewees (e.g. specific aspects of their AF symptoms, impact, experience and views of TREAT). T1 interviews and recordings will be reviewed prior to each T2 interview.**

*Catch up with participant since interview at T1*

1. How have you been feeling since we last met?
2. Has anything significant changed since we last talked e.g. AF symptoms, treatment, INR control, impact of AF

*Behaviour change and the TREATS-AF intervention (section aiming to explore longer term experience of TREATS-AF and maintenance of behavioural modifications; and how this has impacted on them and those around them) – much of this section will relate to discussions at the first interview and therefore may be participant specific.*

1. When we last met we talked about the things that you were being asked to do specifically as part of TREATS-AF (give examples from interview and TREATS-AF). Have you managed to sustain these since we last met?

   Probe – if yes – how? Why?

   If no – reasons and barriers to sustaining change

   Regardless of answers probes can focus on things that underpin or prevent sustainable behaviour change e.g. the TREATS session and resources; interaction with healthcare professionals as part of TREATS or on other occasions; information provision and knowledge requirements; ability / capability to make changes; motivation for change; environmental factors that facilitate or constrain change; social factors (inc. family / friends) that facilitate or constrain change; cost implications of change.
2. Are there any lifestyle changes that have been particularly difficult to implement and / or sustain (depending on responses above)? Again prompt based on responses.
3. Have you used the resources that you brought home with you since we last met? Have these been helpful – if so how, why?
4. Thinking back again about the original TREATS-AF session that we talked about last time – how useful has that been during the last 6 months? Any aspect in particular?
5. Do you feel there is anything that could be improved at all?

*Closing*

1. That’s all the questions I had for you. Before I turn the recorder off, is there anything I haven’t covered that you would like to discuss?

*Closing comments*

- Switch off recorder
- Thank participant for time and ideas
- Re-iterate confidentiality
