# Supplementary material for: Qualitative study of patients’ and clinicians’ experiences of an educational intervention for warfarin therapy control in atrial fibrillation in Thailand
Source: BMJ Open. 2025 Mar 13;15(3):e096490. doi: 10.1136/bmjopen-2024-096490 (PMC11907032; doi:10.1136/bmjopen-2024-096490)
Supplement: online supplemental file 3 [file bmjopen-15-3-s003.docx]

**แนวทางการสัมภาษณ์กลุ่มผู้ให้บริการ (interview guide)**

Objective To understand healthcare professionals’ perspectives regarding their experience and

implementation of the TREAT intervention, including views on the cultural transferability of TREAT to the Thai context

1) สอบถาม อายุ การศึกษา ตำแหน่ง ประสบการณ์ทำงาน (ask about age, education, position and relevant experience in AF treatment)

2) สอบถามว่า ท่านได้สนใจเข้ามามีส่วนร่วมเป็นผู้ให้บริการในโครงการ TREAT-AF ได้อย่างไร (How did you become interested in taking part in the TREATS-AF study?)

2.1) สอบถามเชิงลึก (probe) เกี่ยวกับ การได้มาเป็นทีมให้บริการและแรงบัลดาลใจที่ได้มาร่วมโครงการในฐานะผู้ให้บริการ (Probe around how they found out about the study and what motivated them to take part as a provider in the AF study)

3) สอบถามขั้นตอนการทำ TREAT-AF session ว่าท่านต้องทำอะไรบ้าง (Can you describe what happened on the day of the TREATS-AF session? what was discussed, who delivered the day, what other people were there, what was it like for you, what did you think about it?)

4) ท่านคิดว่าวัตถุประสงค์ของการให้ TREAT-AF คืออะไร (Can you tell me what you think the aims of the TREAT-AF are?)

5) การใช้โปรแกรมนี้ต่างจากรูปแบบการดูแลแบบเดิมอย่างไร (How does TREAT-AF differ from your routine care

5.1 Probe 3 ด้าน : Provider, Patients and Family, service System:

6). อะไรคือข้อดีของ TREAT-AF (What would you say are the good things about it?)

7). อะไรที่ TREAT-AF ควรพัฒนา (Would you say there are any bad things about it?)

8) หากลงรายละเอียดของโปรแกรมแล้ว ซึ่งประกอบด้วย booklet, DVD, worksheet และ diary ท่านทราบได้อย่างไร ว่าคนไข้กำลังตอบสองต่อมัน (How would you know that TREATS-AF is working for your patients? )

8.1 Probe ทำไมถึง work และทำไมอาจไม่work และควรประเมินอย่างไร (why each component may work or may not work and how do they know)
