# Supplementary material for: Qualitative study of patients’ and clinicians’ experiences of an educational intervention for warfarin therapy control in atrial fibrillation in Thailand
Source: BMJ Open. 2025 Mar 13;15(3):e096490. doi: 10.1136/bmjopen-2024-096490 (PMC11907032; doi:10.1136/bmjopen-2024-096490)
Supplement: online supplemental file 4 [file bmjopen-15-3-s004.pdf]

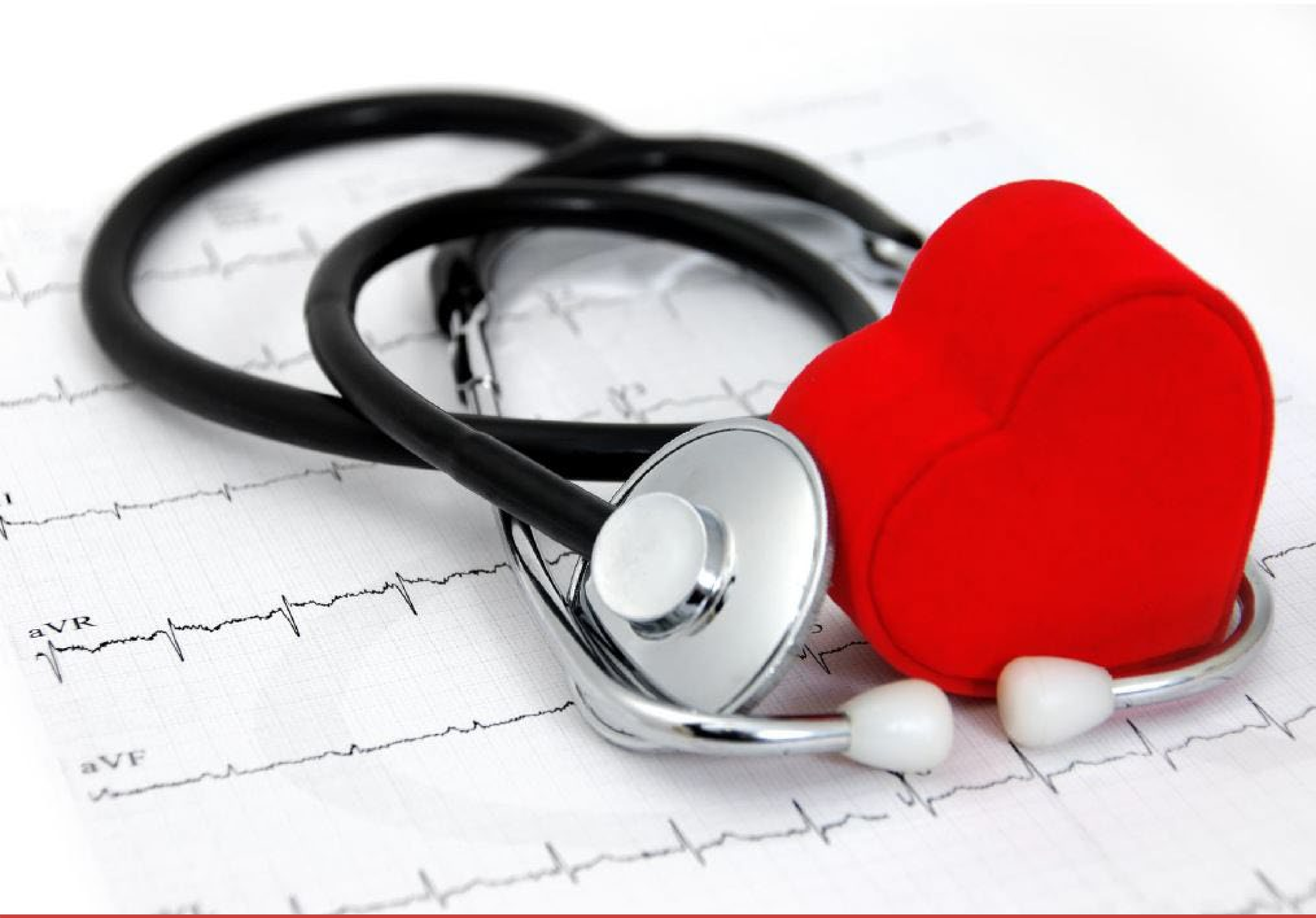

Atrial Fibrillation

Patient Information for treatment with Warfarin

What is atrial fibrillation?

- It is a condition that occurs when electrical impulses in the upper chambers of the heart (atrium) are irregular.
- Consequently, the heart is not systematically functioned (an irregular heartbeat) and the efficacy of blood pumping is reduced.
- People with atrial fibrillation may develop some symptoms e.g. irregular heartbeat, shortness of breath, chest discomfort, fatigue, lightheadedness or fainting.
- Some people with atrial fibrillation may not show any symptom and the conditions are discovered by routine health checkups when they meet general practitioners or are admitted to the hospital due to other conditions.

What are the causes of atrial fibrillation?

- Atrial fibrillation is an age-related condition. There is an increased risk of atrial fibrillation when age increases (shown by the figure below).
- The risk of having Atrial fibrillation is higher in men
- Other conditions or diseases that may increase the risk of atrial fibrillation include stroke, hypertension, diabetes, congestive heart failure, over function of thyroid gland (hyperthyroidism), congenital heart disease (since childhood), and mitral valve disease.
- Overconsumption of alcohol and drug abuse may also increase the risk of atrial fibrillation. However, many people develop atrial fibrillation for no explainable reason.

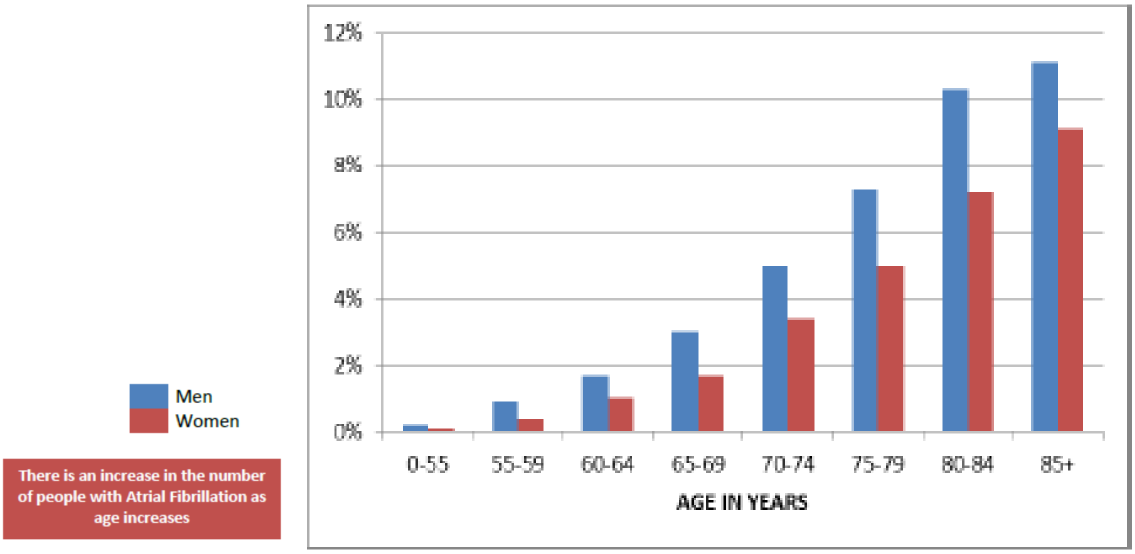

Are there different types of atrial fibrillation?

Yes, there are several types of atrial fibrillation which may occur temporarily in some patients (intermittently) and persistently in other patients.

Five types of atrial fibrillation

1. First diagnosed atrial fibrillation: all patients are first classified into this type, regardless of severity or length of atrial fibrillation.
2. Paroxysmal atrial fibrillation: The atrial fibrillation occasionally occurs whereby each episode is less than 48 hours and then ceases on its own.
3. Persistent atrial fibrillation: This condition lasts longer than 7 days or ceases when treated.
4. Long standing persistent atrial fibrillation: It lasts longer than 1 year, but the patient wants to have the heartbeat returned to a normal heart rhythm of the sinus node.
5. Permanent atrial fibrillation: This condition lasts longer than 1 year and it is the patient and doctor decide to continues this permanent atrial fibrillation.

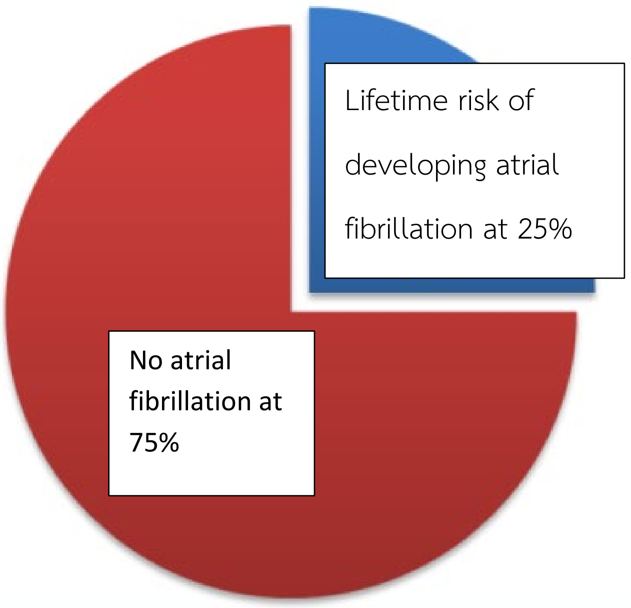

The risk of developing atrial fibrillation is 1 in 4 among people aged 40 years and older; therefore 25% of people will develop atrial fibrillation in their lifetime.

Why are people with atrial fibrillation required to have thinner blood?

- Patients with atrial fibrillation have un-coordinated electrical signals in their upper chambers of the heart.
- This condition causes the heart's chambers to quiver instead of properly pumping blood.
- When the chambers of the heart are not properly functioned, some blood may collect in the upper chamber forming a clot. This clot may travel to any part of the body. It may also cause a stroke if it travels to the brain.
- Reducing blood clots can minimize the risk of patients having a stroke in the future.

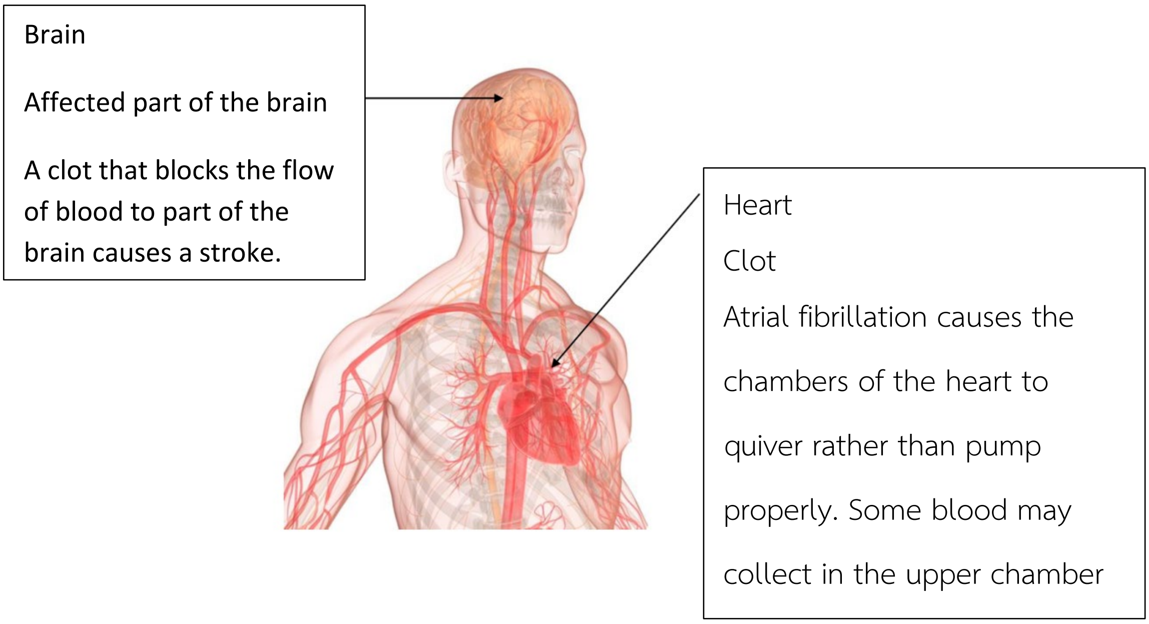

.

Your risk of developing a stroke

One of the methods for assessing your risk of stroke is to give 1 point to each risk factor mentioned below, except some risk factor give 2 points. The higher scores mean the higher risks of stroke. The total score range from 1-9 and the details are shown in the table below.

Assessment on the risk of your stroke

Have you ever had congestive heart failure?	1 Point
Do you have hypertension?	1 Point
Is your age equal to or more than 75 years?	2 Points
Do you have diabetes?	1 Point
Have you ever had a stroke before (even a mild-stroke)?	2 Points
Do you have any vascular diseases (one or more than one of the followings: heart attack, deep vein thrombosis, and atherosclerosis)?	1 Point
Is your age between 65-74 years?	1 Point
Are you female?	1 Point
Total scores	

Suggestions for treatment based on your total risk factor scores

A low risk of a stroke: If you are male and get a score of 0 or your only risk factor is being female (equal to 1 Point), you are not required to take anticoagulants.

A high risk of a stroke: If you are male and get a score of 1 Point or more or if you are female and have other risk factors (see the table above), you will be advised to take anticoagulants: warfarin (INR = 2.0-3.0) or any of new anticoagulants (e.g. dabigatran, rivaroxaban, apixaban or edoxaban).

What are the risks of stroke?

If you are diagnosed with atrial fibrillation but with no other risks of stroke, it is considered that you have a low potential risk. The chance of having stroke less than 1 person in 100 patients (0.78%) in the next year.

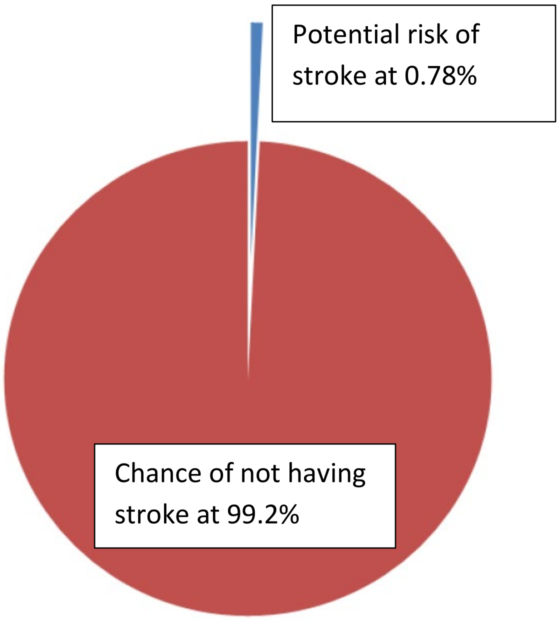

If you are male diagnosed with atrial fibrillation and have another 1 risk factor, it is considered a high risk. The chance of having stroke is 2 people in 100 patients (2%) in the next year if you have not treated.

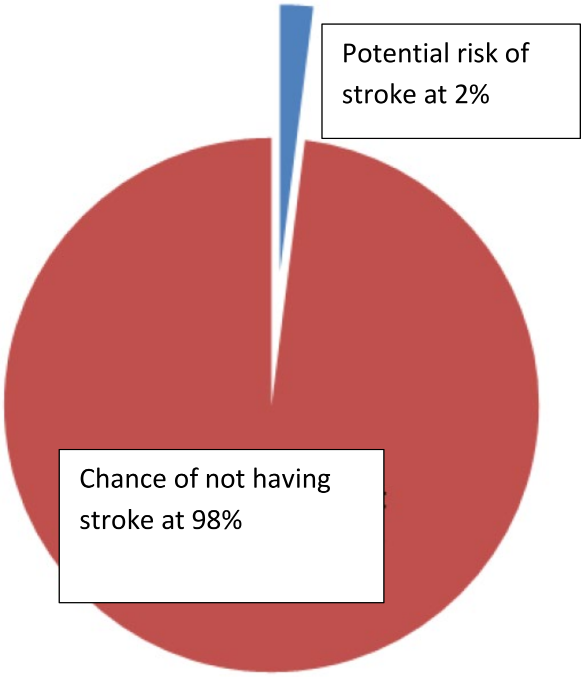

If you (both male and female) are diagnosed with atrial fibrillation and more than two risk factors of stroke, you are considered at a high risk. The chance of having stroke up to 9 people in 100 patients in the next year if you have not treated.

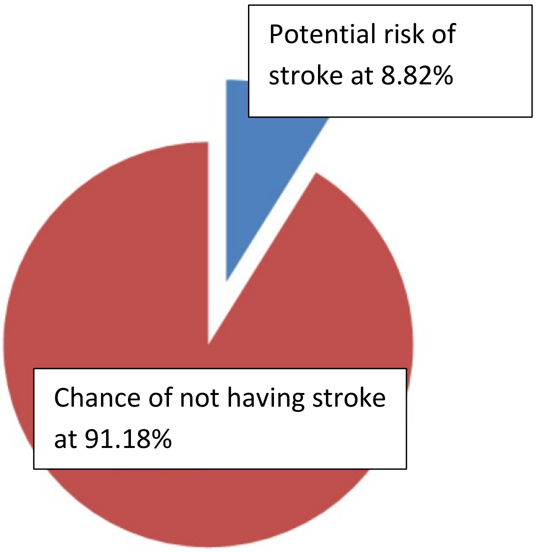

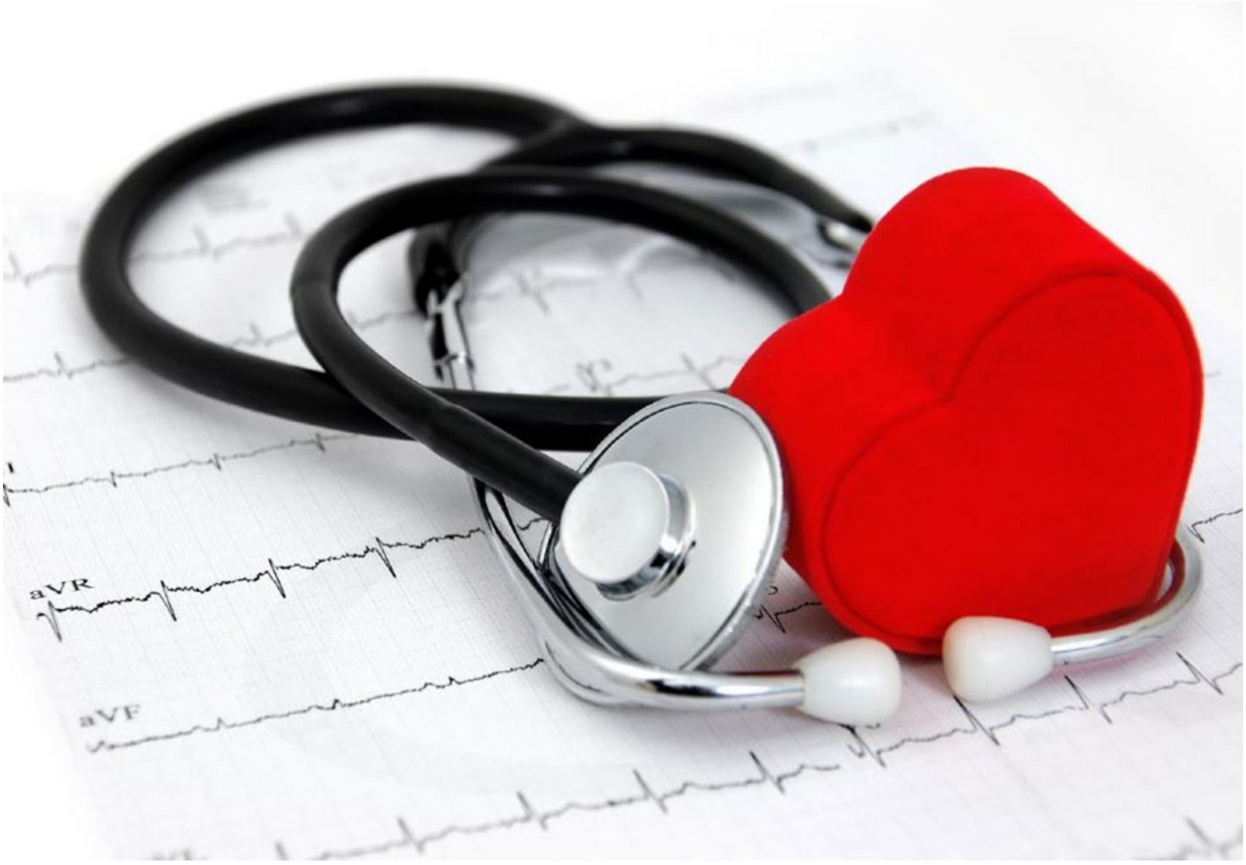

Reducing your risk of stroke

Reducing the risk of stroke in atrial fibrillation with high risk patient is making blood thinner by treating with anticoagulants drug which can prevent stoke more than 60 percentages. It is an efficient drug. Some patients will still have stroke although receiving anticoagulants drug, but only a small number of them (0.2%).

Why can drugs like warfarin make blood thinner?

A clot is caused by two major components in blood.

1. Fibrin: a long protein that binds together to form a mesh
2. Platelets: tiny cell particles that stick to the mesh and help to hold it together

The blood can be thinned to different degrees through the management of fibrin (mesh) or platelets (blood cells).

Drugs like warfarin and heparin are anticoagulants to inhibit the formation of fibrin; therefore they make the blood thinner.

Aspirin and clopidogrel are known as antiplatelet agents that reduce the formation of platelets and prevent blood clots. These drugs do not actually treat atrial fibrillation and they should not be taken together with warfarin unless a doctor prescribes it due to other complication.

Other four blood-thinning medications called an anticoagulant which is not anti-vitamin K drug (NOAC) are now available for medical personnel to prescribe to the patients with atrial fibrillation: dabigatran (Pradaxa), rivaroxaban (Xarelto), apixaban (Eliquis), and edoxaban (Lixina/Savaysa). These drugs are alternatives to warfarin for the same purpose.

What can warfarin do?

- Warfarin acts on the liver and prevents the formation of other substances in blood to create fibrin (mesh of blood cells). This activation makes blood thinner than that of other people in general.

- People are slightly different with the ability to remove warfarin from the body due to difference in ages, body sizes, genders, food, and amount of alcohol intakes. As a

result, each person requires different dosages of warfarin. It is the reason why a dose of warfarin is to be periodically adjusted, for example, when other drugs are added and when there are changes of alcohol intake and food consumption like going on vacation.

What are the risks of warfarin?

- **Severe bleeding:** bleeding occurs in major parts or organs (e.g. brain, spinal cord, eyes, and muscles) which may lead to death or bleeding that requires blood transfusion or alteration of therapy on anticoagulants.
- **No severe bleeding:** bleeding is not severe but may require a hospitalization.
- **Minor bleeding:** other bleeding beyond the extent of conditions mentioned above including minor wounds and ecchymosis.

During 10 years, patients with atrial fibrillation who depend on warfarin will have potential risks of warfarin-related bleeding at approximately 3 in 100 patients

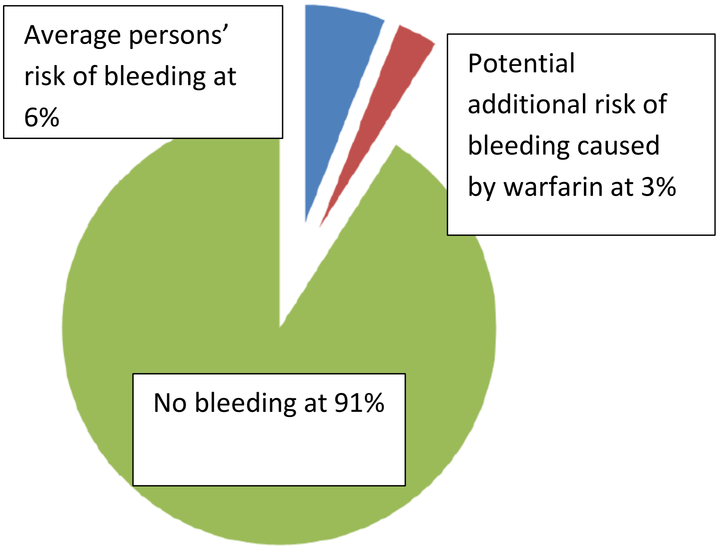

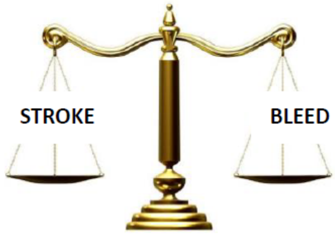

Your doctor needs to weigh your risk of having a stroke and your risk of bleeding before deciding whether or not to give you some medicine to thin your blood. If your doctor feels that you will benefit from taking a blood-thinning medicine, then they will recommend warfarin.

What is INR?

- The efficacy of warfarin is measured by the international normalized ratio (INR), which compares the speed of blood clotting.
- Normal blood coagulation bears an INR of 1. To prevent the risk of stroke in patients with atrial fibrillation, blood must be 2-3 times thinner than normal. It means that blood coagulation will take more time (2-3 times) than that of normal people e.g. an INR range of 2.0-3.0.
- Patients with atrial fibrillation have the desired INR target range of 2.0-3.0.

Warfarin Clinic

- By measuring your INR, the clinic can ensure that your blood will be thinned to the proper ranges.
- If blood is too thick (INR below 2), it shows that you still have a higher risk of stroke than it should be.
- If blood is too thin (INR higher than 3), it may increase potential risks of heavy bleeding in case of wounds or ecchymosis when you fall down. In extreme cases, it may result in severe bleeding (internal bleeding).

How often do I need to attend Warfarin Clinic?

- Once you start taking warfarin, you must attend Warfarin Clinic every week, so they can adjust the dose to suit you.
- Most patients find that after taking warfarin persistently for 2-3 weeks, their INR is relatively stable. Afterwards, they need only attend Warfarin Clinic every 4-8 weeks. However, certain things may affect warfarin and result in the instability of your INR.
- Warfarin Clinic will inform you about the time to attend.

INR control and bleeding prevention

You can do several things to stabilize your INR.

- Control yourself for moderate alcohol intakes: the recommended amount is no more than 1-2 units per day.
- Inform your pharmacist and doctor that you are taking warfarin to ensure that the medications and vitamins are compatible with warfarin.
- Maintain the level of vitamin K in your food (eating similar kinds of food for your food)

Alcohol intake

- If you drink too much alcohol while taking warfarin, you are at an increased risk of complications caused by bleeding.
- You should inform your doctor/pharmacist directly about the amount of alcohol you drink each day and the changes while taking warfarin.
- You should follow up on your alcohol consumption while taking warfarin and should drink no more than 1-2 units per day. Pictures in the next pages will show you how many units of alcohol are contained in each type of beverages.

What is a unit of alcohol?

1 standard drink = Alcohol 10 grams, the amount of alcohol depend on the beverages type that difference of strength of alcohol. The detail of standard drink in favorite beverages is shown in the picture below.

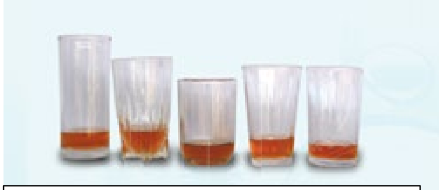

Rice whisky 35-40 degree
30 cc = 1 standard drink

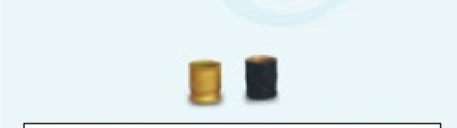

Rice whisky 35-40 degree
50 cc = 1 standard drink

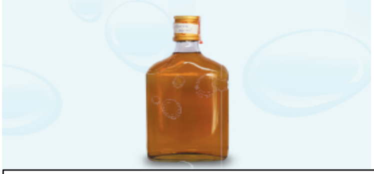

A flat bottle of whisky
volume 350 cc
¼ flat bottle=3 standard drink
½ flat bottle=6 standard drink

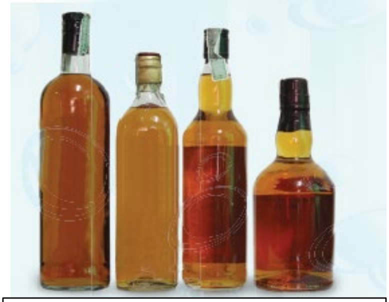

A bottle of whisky
volume 700 cc
¼ bottle=6 standard drink
½ bottle=12 standard drink
1 bottle=24 standard drink

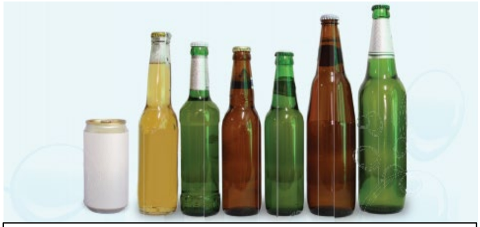

5% beer; Singha, Heineken, Leo, Tiger, Chang draught etc.
¾ can/small bottle=1 standard drink
1 big bottle 660 cc=2.5 standard drink

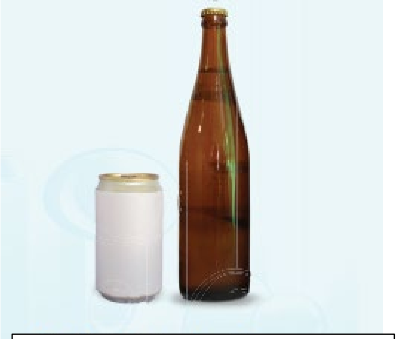

6.5% beer; Chang:
½ can or 1/3 bottle=1 standard drink

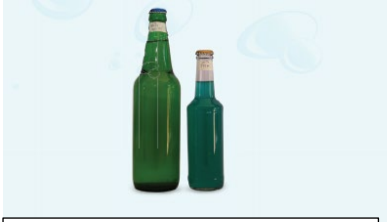

Rice win, local yin:
200 cc=1 standard drink

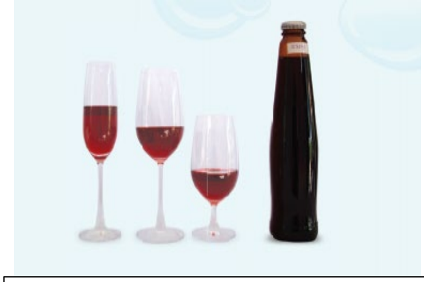

12% Wine:
1 glass 100 cc=1 standard drink
Sparkling wine 1 bottle=1 standard drink

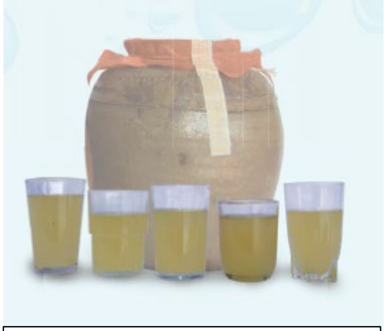

10% of Nam-khao, Krachae
150 cc=1 standard drink

Medications and vitamins

- You should consult with your doctor and pharmacist every time before starting new drugs or vitamins and make them aware that you are now taking warfarin.
- Certain kinds of drugs, herbs, and vitamins can interfere with warfarin. This reaction may result in the activation of warfarin or drug. If it actually affects warfarin, its dose may be accordingly adjusted (more or less) to maintain an INR level within the target range of 2.0-3.0.

Some frequently used pain relievers or over-the-counter medications, such as aspirin and non-steroidal pain relievers like ibuprofen (Brand name; Brufen, Nuprofen) and naproxen (Brand name; Synflex) while can increase the activation of anticoagulation (blood thinning) of warfarin. This may result in blood thinning and pose a potential risk of bleeding.

Do not take the following drugs unless prescribed by doctors.

Drug group	Original name (Thai-English)	Trade name (Thai-English)
1. Other blood-thinning drugs	aspirin	Aspent
	dipyridamole	Persantine
	clopidogrel	Plavix, Apolet
	ticlopidine	Ticlid
2. Non-steroidal pain relievers	ibuprofen	Nurofen
	diclofenac	Voltaren
	piroxicam	Feldene
	mefenamic acid	Ponstan
	celecoxib	Celebrex
	etoricoxib	Arcoxia
3. Other pain relievers	paracetamol*<br>* limit not more than 2 gram if necessary	Sara<br>Tylenol
	tramadol	Tramol
4. Antibiotics	ciprofloxacin	Cipro
	levofloxacin	Cravit
	clarithromycin	Klacid
	erythromycin	E-mycin
	sulfonamide	Bactrim
	cephalosporins	Many trade name
	tetracycline	TC mycin
4. Antibiotic (continue)	cloxacillin	Cloxa)
	dicloxacillin	Dicloxa
	amoxicillin	Amoxcil, Augmentin
	rifampicin	Rifater
	ketoconazole	Nizoral
	itraconozole	Sporal
	fluconazole	Diflucan
	metrodinazole	Flagyl
5. other medication	Seizure medication, antipsychotic drugs, antithyroid agent etc.	

If you have any enquiries about medications you are taking or new drugs prescribed by your doctor whether or not those drugs affect warfarin, please ask your doctor or pharmacist.

Food

- Some food may affect the responses of your body to warfarin.
- It is very essential that you eat the kinds of food similar to those when you first start taking warfarin.
- The dose of warfarin is actually adjusted to be compatible with the food you regularly eat.
- Vitamin K plays a role in normal blood clotting and taking a great amount of Vitamin K will reduce the efficacy of warfarin.
- The main point is that eating the same portion of food containing Vitamin K daily is recommended.
- You must avoid eating food with high Vitamin K such as green leafy vegetable, salad, and soymilk product.
- If you intend to lose weight, eat food with low fat, or change to vegetarian food, your doctor must be informed about these changes for warfarin dosage adjustments.
- It is important that you eat healthy and balancing food. You can eat a small portion of food with moderate to high Vitamin K. You do not have to avoid foods listed on the opposite page.

Please see the table on the following page to help you choose your foods.

Table: International food

Food with low Vitamin K	Food with high Vitamin K	
Artichoke	Avocado	Leaf Mustard
Asparagus	Amaranth	Natto (Fermented Soybeans)
Banana	Broccoli	Black Nightshade
Nut	Brussels Sprout	Okra
Beetroot	Cabbage	Olive Oil
Carrot	Canola Oil	Parsley
Cauliflower	Chayote	Purslane
Celery	Chives	Seaweed
Coriander	Cloeslaw (finely-shredded raw	Soybean Oil
Corn	cabbage with mayonnaise)	Soybean
Eggplant	Kale	Spinach
Mung Bean	Leaves and Stalks of Coriander	Swiss Chard
Capsicum	Endive	Tea from Tonka Bean
Mushroom	Fish in Oil	Tomato
Onion	Chickpea	Turnip
Parsnip	Green Cabbage	Tziton
Pea	Green Tea	Mixed Vegetable Juice (V 8)
Peeled Cucumber	Curly Kale	Watercress
Potato	Kiwi	Wheatgrass Powder
Pumpkin	Lentil	
Radish	Lettuce	
Purple Cabbage	Liver	
Yellow Zucchini	Mayonnaise	
Sweet Potato	Mint Leaves	
Tomato		
Turnip		

Table: Thai food, herb and Thai fruit that should limit or avoid

Food with high Vitamin K	Herb/fruits that should avoid because of disturb of warfarin action
East India spinach	Drumstick
Bog choy	Ginkgo leaves
Piper sarmentosum Roxb	Garlic extract
White Popinac	Ginseng
Water mimosa	Chinese herb
Chinese broccoli	Ginger
water spinach	Bolus
Climbing Wattle	Curcumin
	Dong quai
	Gotu kola
	Jewel Vine
	Derris
	Kariyat
	Many of ripe mango

It is very important that you eat food on a regular basis. Call your doctor or pharmacist if you cannot eat for several days or if you have persistent diarrhea, vomiting, or fever.

Thank you for your participation in this research. If you have any questions regarding this research or the information you receive, please do not hesitate to contact the researchers at Northern cardiac center, Faculty of Medicine, Chiang Mai University

Assoc.Prof.Arintaya Phrommintikul or Miss Siriluck Gunaparn
Northern Cardiac Center, 8th Floor Sriphat Building Faculty of Medicine, Chiang Mai University
Muang Chiang Mai, Chiang Mai 50200
T: 086-923-9142, 089-700-5044 Fax: 053-289-177
Email: sgunaparn@gmail.com

For additional information and suggestions, the following websites and telephone numbers are available for your references.

Thai website

The Heart Association of Thailand under the Royal Patronage

http://www.thaiheart.org/

The association aim to promote, inform and develop the cardiovascular guideline for Thai population.

Contact by phone or website

Telephone number: 02-718-0060-5 Fax number: 02-718-0065 (For telephone please contact during office hours)

Drug Information Center, Faculty of Pharmacy, Mahidol University

https://pharmacy.mahidol.ac.th/dic/

The center is under the supervision of Faculty of Pharmacy, Mahidol University. Free service to answer the question of medication. Contact by phone or website.

Telephone number: 02-644-8685 Fax number: 02-354-4325 (For telephone please contact during office hours)

Herb information office, Faculty of Pharmacy, Mahidol University

http://www.medplant.mahidol.ac.th/index.asp

The center is under the supervision of Faculty of Pharmacy, Mahidol University. Free service to answer the question of herb. Contact by phone or website.

Telephone number: 02-354-4327 or 02-644-8685 # 5305, 5316 (For telephone please contact on office hour)

.

English website

AntiCoagulation Europe (UK)

AntiCoagulation Europe (ACE) is a registered charity and commits itself to prevent blood clotting and provide information and support to people who take anticoagulants or antiplatelet therapy. The information displayed on the website covers quarterly journals, leaflets, and comment forms.

http://www.anticoagulationeurope.org/

Atrial Fibrillation Association (AFA)

Atrial Fibrillation Association (AFA) is a registered charity in UK dedicated to heightening awareness of atrial fibrillation through information and other supports to patients and medical personnel related to searches, diagnoses, and managements of atrial fibrillation.

Telephone number for 24-hour service: (01789) 451837

http://www.atrialfibrillation.org.uk/

The British Cardiac Patients Association

The aim of this association is to provide support, assurance, and advice to cardiac patients, their families, and carers whether it be heart attack, angina, cardiac investigations, arrhythmias, stents, implantable cardiac devices or cardiac surgery for bypass, valve replacement, aneurysm, hole-in-the heart, heart to heart and lung transplant. The association is available for free advice and information.

Telephone number for assistance: (01223) 846845

http://www.bcpa.co.uk/

Europe Heart Rhythm Association (EHRA)

The website of afibmatters.org is developed by Europe Heart Rhythm Association (EHRA of ESC). Its aim is to provide clear, reliable information and practical advice to patients with atrial fibrillation, their families, and carers through developing knowledge and understanding toward atrial fibrillation. The association expects that patients who live with this condition to have more effective health management and longer and better life.

http://www.afibmatters.org/

Other useful sources

Suggestions for patients living with atrial fibrillation

http://circ.ahajournals.org/content/117/20/e340

American Heart Association

http://www.heart.org/HEARTORG/

Atrial Fibrillation Association – Australia

http://www.atrialfibrillation-au.org/

Webmd

www.webmd.com/heart/heart-disease-atrial-fibrillation-basics

Stopafib

www.stopafib.org/

Stopstroke

http://www.stopstroke.com/

Patient.co.uk

www.patient.co.uk/

NHS

www.nhs.uk/Conditions/Atrialwww.nhs.uk/Conditions/Atrial-fibrillation/Pages/Treatment.aspx

Anticoagulation Self-Monitoring Alliance

www.acsma.org.uk/

Daily Treatment Record

How can you complete your daily treatment record?

Thank you for your participation in our research. Here are some points you must be aware of when filling out your daily treatment record. Please keep in mind that this is your daily record. Data recording can ensure you that your warfarin therapy will be more successful by helping you to find out factors excluding your INR from the desired target range.

- You should write down your daily record during the first two weeks after you participate in the educational intervention and start taking warfarin.
- Please do not be over-concerned about the perfect spellings, grammatical structures, or handwriting, but try to write as clearly as possible by using a pen.
- You should write down your daily record every evening. If you cannot do so for any day, record it on the following day.
- If you feel that you have missed your recording for several days, please do not give up for the remaining days in that week. Start recording it again on the following day you can do and leave the remaining pages empty.
- Please refer to a booklet giving information about warfarin regarding the section displaying the pictures of several kinds of alcohol units. Do not worry if you cannot find the certain units of beverages you drink.
- Record about staple food you eat daily e.g. toast with marmalade, chicken salad with dressing, baked beef with broccoli, carrot, and potato. Try to record kinds of vegetables you eat, including fruit juice or snack, particularly fruit or vegetable juice.

Everything written down on your daily record may affect your target INR. If your INR is not within the proper range, you may have an increased risk of stroke or bleeding. By controlling the volume of your alcohol, food and drug, your warfarin therapy will be successful in reducing the risk of stroke or severe bleeding. When you write down your daily record each week, think about your lifestyle in that week (e.g. food, alcohol, and medications) on how it affects your INR.

Week 1: Starting Date: ____/____/_____

	Recording food, fruit, vegetables, and snacks you eat today	Recording all alcoholic beverages you drink today	Units per day	Recording any health problems observable today (e.g. bleeding, ecchymosis, and tiredness)
Monday				
Tuesday				
Wednesday				
Thursday				
Friday				
Saturday				
Sunday				

If you need more space for full recording, please use the treatment record on the back.

Week 2: Starting Date: ____/____/_____

	Recording food, fruit, vegetables, and snacks you eat today	Recording all alcoholic beverages you drink today	Units per day	Recording any health problems observable today (e.g. bleeding, ecchymosis, and tiredness)
Monday				
Tuesday				
Wednesday				
Thursday				
Friday				
Saturday				
Sunday				

If you need more space for full recording, please use the treatment record on the back.

Medications

Did you forget to take warfarin? Yes____No______

If yes, when? ____

Why did you forget taking it? ____

Do you take any vitamin, dietary supplement, herbal cure, or new drug during this week?

Yes_____ No______

If yes, when? ____

What is its named? ____

INR

Did you have INR measured at hospital or clinic? Yes_____No______

If yes, please answer the following questions. What is your INR?_____________

Is your INR within the range of 2.0-3.0? Yes_____No______

If no, please answer the following questions.

Considerations

If your INR is within the range of 2.0-3.0, do not answer the following questions.

Did you drink a large volume of alcohol on any day of this week?

Did you eat food with high Vitamin K such as green cabbage, watercress, and soybean greater than normal amounts?

(For the lists of all food, please refer to the booklet entitled 'Patient Information on Warfarin Therapy'.

Do you eat food regularly?

Do you start eating/taking new things? These may include prescription drugs or over-the-counter medications, vitamins, or herbs. Please be aware of these and write them down on your weekly record. Please describe the reasons why you fail to reach your target INR?

1. ____

2. ____

3. ____

My Treatment Record

Warfarin Monitoring Table

Part A: Risk assessment of your stroke

One of the methods for assessing your risks of a stroke is through CHA_2DS_2-VASc. For scoring CHA_2DS_2-VASc, give 1 point to each risk factor mentioned below, except for stroke and age equal to or more than 75 years which are given two points. The higher scores mean the higher risks of stroke.

Calculate the risks of your stroke by answering each question in the table below. If any questions are inapplicable to you, put 0 into your score blank.

Questions	Score	Your score
Is your age equal to or more than 75 years?	2 Points	
Do you have hypertension?	1 Point	
Do you have diabetes?	1 Point	
Have you ever had congestive heart failure?	1 Point	
Have you ever had a stroke before (even a mile-stroke)?	2 Points	
Do you have any vascular diseases (one or more than one of the followings: heart attack, deep vein thrombosis, and atherosclerosis)?	1 Point	
Is your age between 65-74 years?	1 Point	
Are you female?	1 Point	
Total scores		

Suggestions based upon your total scores

A very low risk of stroke: If you are male and get a score of 0 or your only risk factor is being female (score equal to 1 Point), you are not required to take anticoagulants.

A risk of stroke: If you are male and get a score of 1 Point or more or if you are female and have other risk factors (see the table above), you will be advised to take anticoagulants: warfarin (INR = 2.0-3.0) or any of new anticoagulants (e.g. dabigatran, rivaroxaban, apixaban or edoxaban).

Part B: Your warfarin therapy plan

What is your target INR? ____

Lifestyle adjustments:

Target volume of daily alcoholic beverages ____________________Units

Alcoholic beverages you drink on a regular basis

1. ___________________________________ Units ________________

2. ___________________________________ Units________________

3. ___________________________________ Units ________________

How many days per week do you drink alcoholic beverages? _____________________

Part C: Concerns about medication

Once patients are required to take their lifelong prescriptions e.g. warfarin, they may develop some problems or concerns inhibiting an intake of medications and are not benefited from reducing the risk of stroke. These are probably psychological concerns such as concern about side effects, drug burden, or concern about practices i.e. how to remember to take drugs and what to do if they forget to take drugs, etc.

In the space below, please describe your concerns about taking warfarin:

____

____

____

____

____

____

You should consult your doctor and medical personnel regarding these concerns every time. You can bring these topics into your group discussion. Alternatively, you can contact any researcher on the following day if you would like to talk about these concerns privately.

Assoc.Prof.Arintaya Phrommintikul or Miss Siriluck Gunaparn

Northern Cardiac Center, 8th Floor Sriphat Building Faculty of Medicine, Chiang Mai University

Muang Chiang Mai, Chiang Mai 50200

T: 086-923-9142, 089-700-5044 Fax: 053-289-177

Email: sgunaparn@gmail.com
