# Supplementary material for: Qualitative study of patients’ and clinicians’ experiences of an educational intervention for warfarin therapy control in atrial fibrillation in Thailand
Source: BMJ Open. 2025 Mar 13;15(3):e096490. doi: 10.1136/bmjopen-2024-096490 (PMC11907032; doi:10.1136/bmjopen-2024-096490)
Supplement: Supplementary file 5 [file bmjopen-2024-096490.reviewer_comments.pdf]

PEER REVIEW HISTORY

BMJ Open publishes all reviews undertaken for accepted manuscripts. Reviewers are asked to complete a checklist review form and are provided with free text boxes to elaborate on their assessment. These free text comments are reproduced below.

ARTICLE DETAILS

Title (Provisional)

Qualitative study of patients' and clinicians' experiences of an educational intervention for warfarin therapy control in atrial fibrillation in Thailand

Authors

Jiraporncharoen, Wichuda; Buawangpong, Nida; Angkurawaranon, Chaisiri; Jolly, Kate; Neil Thomas, G.; Phrommintikul, Arintaya; Krittayaphong, Rungroj; Nathishuwan, Surakit; Lip, Gregory YH; Lane, Deirdre; Mathers, Jonathan; CONSORTIUM, TREATS-AF

VERSION 1 - REVIEW

**Reviewer**	**1**
**Name**	**Zhou, Zien**
**Affiliation**	**Ren Ji Hospital, School of Medicine, Shanghai Jiao Tong University, Radiology**
**Date**	**17-Dec-2024**
**COI**	**None**

Jiraporncharoen et al. reported a qualitative study of the perception of people with lived experience of AF and HCPs regarding the intervention of an education program on AF and anticoagulation management that is being adopted in an ongoing trial. This educational program is interesting. My comments are added below:

1. Why only the management of warfarin is focused on in this educational intervention in the context of NOAC being a first-line recommendation for people with non-valvular AF nowadays? If warfarin management is only involved in this educational program, suggest clarifying in the title.

2.(Page 2, line 21) Please briefly introduce the TREATS-AF intervention in the abstract.

3. The authors mentioned three acronyms – TREAT, SAMe-TT2R2, and TREATS-AF. Please give the full names of them. I was a bit confused about their relationships before reading the method section. Also, please give the full name of TTR on Page 5, Line 13.

4. Although the authors mentioned a limitation of sampling is that a majority of HCPs are pharmacists. Another shortness is that they are all females. Is it due to the focus group being "conducted by one experienced female clinical qualitative researcher"? Is the quota in sex/gender considered in the purposive sampling of potential participants?

5. How were the four themes determined? Generally, I would suggest that the authors concisely describe their results. Figure 2 nicely summarises the pros of this educational program from the view of people with lived experience and HCPs. I suggest adding a summary of the cons that were mentioned in the Results.

6. Is there any quantitative data in the last theme (e.g. proportion of self-reported behaviour changes)?

7. An agreement to share TREATS-AF educational documents from the authors may help other researchers on this topic.

**Reviewer**	**2**
**Name**	**Scicchitano, Pietro**
**Affiliation**	**ASL Bari, Cardiology**
**Date**	**17-Dec-2024**
**COI**	**None**

- the use of semi-structured interviews might be considered as a limitation. This should be discussed into a dedicated limitation section.

- the small sample size is a limitation of the paper- This should be discussed in a dedicated limitation section. Please discuss such a point.

- introduction is too long. Please reduce its length and be more focused on the aims of the paper.

- there is no mention to the reproducibility of this structured interview. Please discuss.

- Themes and subthemes paragraphs can be moved to supplementary materials

---

VERSION 1 - AUTHOR RESPONSE

Reviewer 1

General comment: Jiraporncharoen et al. reported a qualitative study of the perception of people with lived experience of AF and HCPs regarding the intervention of an education

program on AF and anticoagulation management that is being adopted in an ongoing trial. This educational program is interesting.

Response: We would like to thank the reviewer for the positive comments. Below are our point-by-point responses.

Comment 1) Why only the management of warfarin is focused on in this educational intervention in the context of NOAC being a first-line recommendation for people with non-valvular AF nowadays? If warfarin management is only involved in this educational program, suggest clarifying in the title.

Response to comment 1) At the time of writing the grant and starting the project, warfarin was the only anticoagulant used routinely in clinical practice in Thailand. This is the reason why TREATS-AF focusses exclusively on warfarin. We have changed the title to reflect this. "Qualitative study of patients' and clinicians' experiences of an educational intervention for warfarin therapy control in atrial fibrillation in Thailand".

Comment 2) (Page 2, line 21) Please briefly introduce the TREATS-AF intervention in the abstract.

Response to comment 2) We have added to the abstract to this effect.

Comment 3) The authors mentioned three acronyms – TREAT, SAMe-TT2R2, and TREATS-AF. Please give the full names of them. I was a bit confused about their relationships before reading the method section. Also, please give the full name of TTR on Page 5, Line 13.

Response to comment 3) We would like to apologise for our mistake. We have provided names in full on first mention and given a brief explanation of the SAMe-TT_2R_2 score in the introduction.

Comment 4) Although the authors mentioned a limitation of sampling is that a majority of HCPs are pharmacists. Another shortness is that they are all females. Is it due to the focus group being "conducted by one experienced female clinical qualitative researcher"? Is the quota in sex/gender considered in the purposive sampling of potential participants?

Response to comment 4) Quota sampling for gender was not used for providers. In our setting, pharmacists are primarily responsible for teaching patients how to use warfarin, and most pharmacists in Thailand are female. We have added this explanation to our discussion (page 29).

Comment 5) How were the four themes determined? Generally, I would suggest that the authors concisely describe their results. Figure 2 nicely summarises the pros of this educational program from the view of people with lived experience and HCPs. I suggest adding a summary of the cons that were mentioned in the Results.

Response to comment 5) The four themes were determined via the analytical process using the framework approach as described in the data analysis section. At the end of this section we indicate that the analysis (and hence themes) were broadly organized with respect to the research questions focused on the experience of the TREATS-AF intervention.

Comment 6) Is there any quantitative data in the last theme (e.g. proportion of self-reported behaviour changes)?

Response to comment 6) We did not collect quantitative data relating to the proportion of self-reported behaviour change in the study.

Comment 7) An agreement to share TREATS-AF educational documents from the authors may help other researchers on this topic.

Response to comment 7) We have added the translated version of the educational booklet as supplementary material to the paper.

Reviewer 2

Comment 1) the use of semi-structured interviews might be considered as a limitation. This should be discussed into a dedicated limitation section.

Response to comment 1) Semi-structured interviews are a very common qualitative data collection method allowing research participants to describe in-depth their experiences and

perspectives, and allowing a participant-focused data collection approach. This is a key facet of qualitative research and one which we felt was appropriate to explore participants' experiences of the TREATs-AF intervention. Without further information about the potential limitations of this data collection method inferred by the Reviewer's comments it is difficult to discuss further. Semi-structured interviews, as opposed to structured approaches, are used in qualitative research to ensure that data collection is participant focused and that researcher perspectives and biases are not replicated, for instance as might be the potential in structured or fixed (quantitative) interview approaches.

Comment 2) the small sample size is a limitation of the paper- This should be discussed in a dedicated limitation section. Please discuss such a point.

Response to comment 2) Please see our response to Editor's comment where we have provided more justification for our sample size and approach in the Methods section. We have also included discussion of the limitations of the sample achieved e.g. breadth of provider interviews from the wider multidisciplinary team. We would note that smaller sample sizes are common in qualitative research that privileges depth over breadth (across a large sample) of insight versus quantitative approaches such as survey research.

Comment 3) introduction is too long. Please reduce its length and be more focused on the aims of the paper.

Response to comment 3) We have revised the introduction as per the reviewer's suggestion but note that we have also added a small amount of detail in response to Reviewer 1's comments and requests.

Comment 4) there is no mention to the reproducibility of this structured interview. Please discuss.

Response to comment 4) We have now included copies of our interview schedules that guided the semi-structured interview conduct for all participants. As per our response to the

Editor we have now provided these as supplementary materials to our paper so that a reader can judge how the data were produced via the qualitative interviews.

Comment 5) Themes and subthemes paragraphs can be moved to supplementary materials

Response to comment 5) The descriptive presentation of themes and sub-themes are a core component of the rigorous presentation of qualitative research findings. These paragraphs are a central component of the main paper and, for example, relate to core items in the quality checklist requested by the BMJ Open for judging the reporting of qualitative research (items 29-32 in the COREQ checklist submitted with the paper). Therefore, we would maintain that the themes and sub-themes remain in the main document because they represent the objectives of the study and the main findings that address these questions.
